# Stereotactic body radiation therapy for ultra-central lung malignancies: an updated systematic review and meta-analysis

**DOI:** 10.1186/s13014-026-02854-5

**Published:** 2026-05-11

**Authors:** Hai Zeng, Xiaofeng Wang, Hui Bai, Wei-Jia Zhang, Jun Cai, Wenli Chen, Yi Qin, Jun Zhang, Yunxiang Qi, Churong Li, Yan-Ling Wu

**Affiliations:** 1 Department of Oncology, The First Affiliated Hospital of Yangtze University, Jingzhou, Hubei 434000 P. R. China; 2 Department of Radiation Oncology, Radiation Oncology Key Laboratory of Sichuan Province, Sichuan Clinical Research Center for Cancer, Sichuan Cancer Hospital and Institute, Sichuan Cancer Center, University of Electronic Science and Technology of China, Chengdu, Sichuan Province China; 3 Department of Radiology, Sichuan Clinical Research Center for Cancer, Sichuan Cancer Center, Sichuan Cancer Hospital and Institute, University of Electronic Science and Technology of China, Chengdu, 610041 China; 4 Department of Medical Oncology, Sichuan Clinical Research Center for Cancer, Sichuan Cancer Hospital and Institute, Sichuan Cancer Center, Affiliated Cancer Hospital of University of Electronic Science and Technology of China, Chengdu, China; 5 Department of Radiation Oncology, National Cancer Center/National Clinical Research Center for Cancer/Cancer Hospital and Shenzhen Hospital, Chinese Academy of Medical Sciences and Peking Union Medical College, Shenzhen, Guangdong 518116 China

**Keywords:** SBRT, Efficacy, Safety, Ultra-central, Lung malignancies

## Abstract

**Purpose:**

Stereotactic body radiotherapy (SBRT) is a highly effective and well-tolerated treatment modality for peripherally located early-stage non-small cell lung cancer as well as pulmonary metastases. However, its feasibility and safety remain uncertain in patients with ultra-central (UC) lung tumors. This study aims to evaluate the efficacy and safety of SBRT in the management of UC lung malignancies.

**Methods:**

A systematic review of the available literature was conducted in accordance with the PRISMA guidelines, incorporating studies that reported both treatment-related side effects and therapeutic efficacy associated with SBRT for UC lung malignancies. Patient characteristics, prescription doses, local control (LC), overall survival (OS), and side effects information were extracted. Clinical outcomes were analyzed using the random-effects model. Meta-regression was conducted to determine the effect of various covariates on LC and OS outcomes.

**Results:**

Twenty-three articles with a total of 1,105 patients were included. Pooled 1- and 2-year LC rates were 93% (95% CI: 88–96%) and 84% (95% CI: 77–89%), respectively. Meta-regression demonstrated a significantly positive association between BED_10_ and 1- and 2-year LC rates (*P* = 0.001, *P* < 0.001, respectively), and a significantly negative association between PTV volume and 1- and 2-year LC (*P* = 0.048, *P* = 0.030, respectively). Pooled 1- and 2-year OS rates were 81% (95% CI: 77–85%) and 58% (95% CI: 52–64%), respectively. Meta-regression revealed a significantly positive association between BED_10_ and 1- and 2-year OS rates (*P* = 0.025, *P* = 0.002, respectively), and a significantly negative association between PTV volume and 1- and 2-year OS (*P* = 0.031, *P* < 0.001, respectively). The pooled rates of grade ≥ 3 and grade 5 treatment-related side effects were 9% (95% CI: 6–14%) and 2% (95% CI: 1–5%), respectively.

**Conclusion:**

This meta-analysis demonstrates that SBRT achieves favorable LC rates and is a relatively safe treatment for UC lung malignancies. Although the pooled rate of grade 5 side effects, at 2%, was within an acceptable range, vigilance remains essential. Further prospective research is warranted to verify our conclusions.

**Supplementary Information:**

The online version contains supplementary material available at 10.1186/s13014-026-02854-5.

## Introduction

Stereotactic body radiation therapy (SBRT) is a well-established treatment modality for patients with inoperable early-stage non-small cell lung cancer (ES-NSCLC) as well as for those with isolated pulmonary metastasis, offering high rates of local control and favorable survival outcomes through the precise delivery of ablative radiation doses to the target lesion [1, 2].

SBRT demonstrates excellent local tumor control with a favorable toxicity profile for peripherally located lung tumors, typically with very low rates of grade 4 or 5 adverse events [3–5]. However, lesions in proximity to the proximal bronchial tree (PBT) and mediastinal structures may pose increased risks of fatal side effect [6, 7]. The remarkably high side effect rates observed in the early phase II trial conducted by Timmerman et al., which utilized a radiation dose of 60–66 Gy delivered in three fractions, revealed that patients with central tumors (within 2 cm of the PBT) had an 11-fold increased risk of severe toxicities, including an elevated risk of treatment-related mortality [8]. Subsequent studies, including the landmark Radiation Therapy Oncology Group (RTOG) 0813 trial, determined that a risk-adapted SBRT approach was safe for central lesions when using a lower fraction size (e.g., 60 Gy in 5 fractions), with grade 3 to 4 side effect rates below 10% and a treatment-related mortality risk below 1% [9, 10]. However, the definition of a “central” tumor location includes a heterogeneous group of patients with differing risks of side effects, and a subset of central tumors that directly abut or invade the PBT, esophagus, trachea, or other mediastinal structures are termed “ultra-central (UC)” and are associated with a substantial risk of severe side effects from SBRT [7, 11–13]. A recent meta-analysis by Yan et al. revealed that the pooled incidence of treatment-related death in SBRT for UC lung tumors was 4%, with the most common cause being hemoptysis [14]. More recently, the HILUS trial delivered a radiation dose of 56 Gy in 8 fractions to UC tumors and reported that the incidence of treatment-related death was 15% [12]. Notably, UC lesions are often either inoperable or necessitate extensive surgical interventions, such as pneumonectomy. Consequently, the feasibility of SBRT, which offers a potential curative option for UC lesions, holds significant clinical importance.

However, although the use of SBRT for UC malignancies is becoming more widespread, radiation fractionation regimens are frequently derived from individual clinical experience, and the safety and efficacy of this approach have not yet been systematically established. To address these gaps, this study conducted a systematic review of existing literature on the use of SBRT for UC lung malignancies, evaluated dose–response relationships, and assessed the efficacy and safety of SBRT in this specific clinical context. Distinct from the prior meta-analysis by Yan et al. [14]., which provided a comprehensive overview of earlier data, this study incorporates findings from recent pivotal prospective trials. Furthermore, we perform quantitative meta-regression analyses to specifically evaluate the impact of PTV volume and biologically effective dose (BED) on local control, providing updated evidence-based information for clinical practice in this high-risk population.

## Materials and methods

### Study design and literature search strategy

This meta-analysis was conducted in accordance with the Preferred Reporting Items for Systematic Reviews and Meta-Analyses (PRISMA) [15], and it aimed to evaluate the efficacy and safety of SBRT for UC lung malignancies. The inclusion criteria were defined according to the Population, Intervention, Comparison and Outcome (PICO) framework, as detailed in Supplementary Table 1. The specific inclusion criteria are as follows: (1) Prospective or retrospective studies with a cohort size of ≥ 10 UC lung malignancies; (2) Adult patients (> 18 years) with UC lung malignancies (primary or metastatic tumors) receiving SBRT at ≥ 5 Gy per fraction; (3) Mandatory reporting of local control rate (LCR), OS or side effects data; (4) Exclusive focus on first-course SBRT (studies including re-irradiation must provide distinguishable outcome data); (5) English-language publications; (6) For duplicate publications, only the most recent/informative report was included. Notably, the definition of “UC” varied based on the specific study, but generally included tumors in which the gross tumor volume (GTV) or planning target volume (PTV) abuts or overlaps the PBT or other mediastinal structures (e.g., trachea, esophagus, heart). Therefore, in this meta-analysis, we define lung tumors with PTV abutting or overlapping the PBT, or other mediastinal structures as UC lesions. Table 1 summarizes the various definitions reported in included studies.

**Table 1:** Baseline characteristics of 23 studies

Study (Year)	Study design	Country	Patients (*n*)	TNM Stage	Tumor Type	Age(y), Median (Range)	Sex (M/F)	Definition of ultra central lung cancer	Follow-up(m), Median (Range)
Unger (2010)	Retrospective	USA	20	NA	Mixed: Primary NSCLC: 15%, Metastases: 85%	64 (13–82)	10/10	GTV abutting/invading mainstream bronchus	10 (NA)
Tekatli (2016)	Retrospective	Netherlands	47	7th	100% Primary NSCLC (Stage I-IIIA: 39 cases; including 8 recurrences)	78 (58–91)	35/12	PTV overlapping trachea/main bronchi	29.3 (95% CI: 21.5–37.1)
Haseltine (2016)	Retrospective	USA	18	7th	Mixed: Primary NSCLC: 94.4%, Metastases: 5.6%	69 (49–87)	9/17	Tumors abutting the PBT	22.7(NA) (Entire cohort)
Lischalk (2016)	Retrospective	USA	20	NA	100% Metastatic	66 (24–82)	10/10	Abutment/invasion of mainstem bronchus	19 (NR)
Raman (2018)	Retrospective	Canada	26	NA	Mixed: Primary NSCLC: 80.8%, Metastases: 15.4%, Unknown: 3.8%	73 (44–89)	9/17	PTV contacting/overlapping proximal PBT, trachea, esophagus, pulmonary vein/artery	21.4 (NA) (Entire cohort)
Cong (2019)	Retrospective	China	51	8th	100% NSCLC (Stage III: 20; Stage IV: 19; Recurrent: 12)	63 (35–82)	33/18	GTV abutting/overlapping trachea/PBT	17 (NA)
Nguyen (2019)	Retrospective	USA	14	NA	Mixed: Primary NSCLC 92.9%, Metastatic: 7.1%	66 (41–87)	9/5	PTV touching PBT/esophagus	16.2 (4.2–72.5)
Regnery (2020)	Retrospective	Germany	51	NA	Mixed: Primary NSCLC: 82.4%, Metastases: 17.6%	70.5 (Mean, SD:11.96)	32/19	PTV overlapping PBT	Not reached (0.9–86)
Cooke (2020)	Retrospective	UK	22	NA	100% Metastatic	71 (38–89) (Entire cohort)	16/11 (Entire cohort)	GTV directly abutting PBT	14.3 (NA)
Yang (2020)	Retrospective	China	21	7th	100% NSCLC (New/Recurrent/Second primary)	66 (52–81)	12/9	PTV abutting/overlapping proximal bronchial tree, heart, great vessels (esophagus excluded)	15 (4–70)
Zhao (2020)	Retrospective	Canada	41	8th	Mixed: Primary lung tumors: 73.2%; Metastases: 26.8%	72 (44–85)	19/22	PTV overlap: proximal bronchial tree/esophagus/pulmonary vein/artery	22.9 (NA) (Entire cohort)
Lindberg (2021)	Prospective	Sweden	65	NA	Mixed: Primary NSCLC: 78%;Metastases: 22%	70 (49–91)	37/28	Tumor localized within 1 cm of the proximal bronchial tree	24 (3–76)
Lodeweges (2021) ^#^	Retrospective	Netherlands	72	7th	Mixed: Primary lung tumors: 76%, Nodal metastases: 24%	72 (IQR 64–79)	45/27	PTV abutment/overlap: main bronchi/trachea/esophagus	19 (IQR 10–32)
Mihai (2021)	Retrospective	Ireland	57	NA	Mixed: Primary NSCLC: 65%, Metastases: 35%	72 (34–85)	31/26	CTV abutting/involving trachea/main/lobar bronchi	26.5 (3.2–100.5)
Breen (2021)	Retrospective	USA	110	NA	100% Primary NSCLC (T1-3N0M0)	75 (50–94)	52/58	GTV touching PBT/trachea; PTV overlap trachea/mainstem bronchi; GTV ≤ 1 cm from PBT	30 (4.8–100.8)
Loi (2021)	Retrospective	Italy	109	NA	Mixed: Primary NSCLC: 66%, Metastases: 34%	70 (28–86)	NA	PTV overlap: central bronchial tree/esophagus/ pulmonary vein/artery	17 (3–78)
Guillaume (2021)	Retrospective	France	74	NA	Mixed: Primary NSCLC: 50%, Metastases: 50%	69 (19–90)	46/28	PTV overlapping with the trachea, right and left main bronchi, intermediate bronchus, lobe bronchi, esophagus, heart	25 (3–86)
Farrugia (2021)	Retrospective	USA	43	8th	100% Primary NSCLC (T1-4N0M0)	75 (IQR 72-77.5)	18/25	GTV abutting the proximal bronchial tree, trachea, mediastinum, aorta, or spinal cord.	23.7 (16.6–32.4)
Wang (2022)	Retrospective	China	58	7th	100% Primary NSCLC (T1-3N0M0)	68 (46–85)	43/15	PTV touching/overlapping proximal bronchial tree, trachea, esophagus, heart, pulmonary vein or pulmonary artery within 2 cm around bronchial tree.	57 (6–90)
Salvestrini (2022)	Retrospective	Netherlands	122	NA	Mixed: Primary NSCLC: 55.7%, Metastases: 44.3%	72 (34–91)	71/51	PTV touches or overlaps the trachea, mainstem-, intermediate-, upper-, middle- or lower- lobe bronchus or the esophagus.	23 (0.3–159.4)
Regnery (2023)	Prospective	Germany	16	NA	Mixed: Primary NSCLC: 25%, Metastases: 75%	66 (IQR 55–72)	9/7	PTV overlap with PBT/esophagus	23.6 (5.4–32.2)
Giuliani (2024)	Prospective	Canada	30	8th	100% Primary NSCLC(T1-3N0M0)	73 (65–87)	13/17	PTV touching/overlapping central bronchial tree, esophagus, pulmonary vein/artery	37 (8.9–51)
Lee (2024)	Retrospective	USA	18	NA	Mixed: Primary NSCLC: 27%, Metastases: 73%(Entire cohort)	66 (35–77) (Entire cohort)	11/15 (Entire cohort)	GTV abutting PBT/esophagus/great vessels	17 (2–34)

Note and abbreviations: ^#^Three patients (4%) had previously received radiotherapy for a prior lesion in the same lung as the index lesion. The interval between their previous radiotherapy and the current SBRT exceeded one year in all three cases, and none experienced grade 3 or higher toxicity. NSCLC: Non-Small Cell Lung Cancer; PTV: Planning Target Volume; IQR: Interquartile Range; NA: Not Available; SD: Standard Deviation; M: male; F: female; PBT: proximal bronchial tree

We systematically searched PubMed, Embase, Cochrane Library, and Web of Science databases from the databases’ inception up to October 1, 2025. The detailed Queries are shown in (Supplementary Table 2). To obtain the latest information, we also searched the abstracts in the American Society of Clinical Oncology (ASCO), American Society for Radiation Oncology (ASTRO), European Society for Radiation Oncology (ESTRO), and the European Society for Medical Oncology (ESMO).

### Study endpoints

The primary endpoints were the 1- and 2-year LCR and the incidences of grade ≥ 3 and grade 5 treatment-related toxicities. Secondary endpoints were the 1- and 2-year rates of OS.

### Study selection and data extraction

**Fig. 1:**
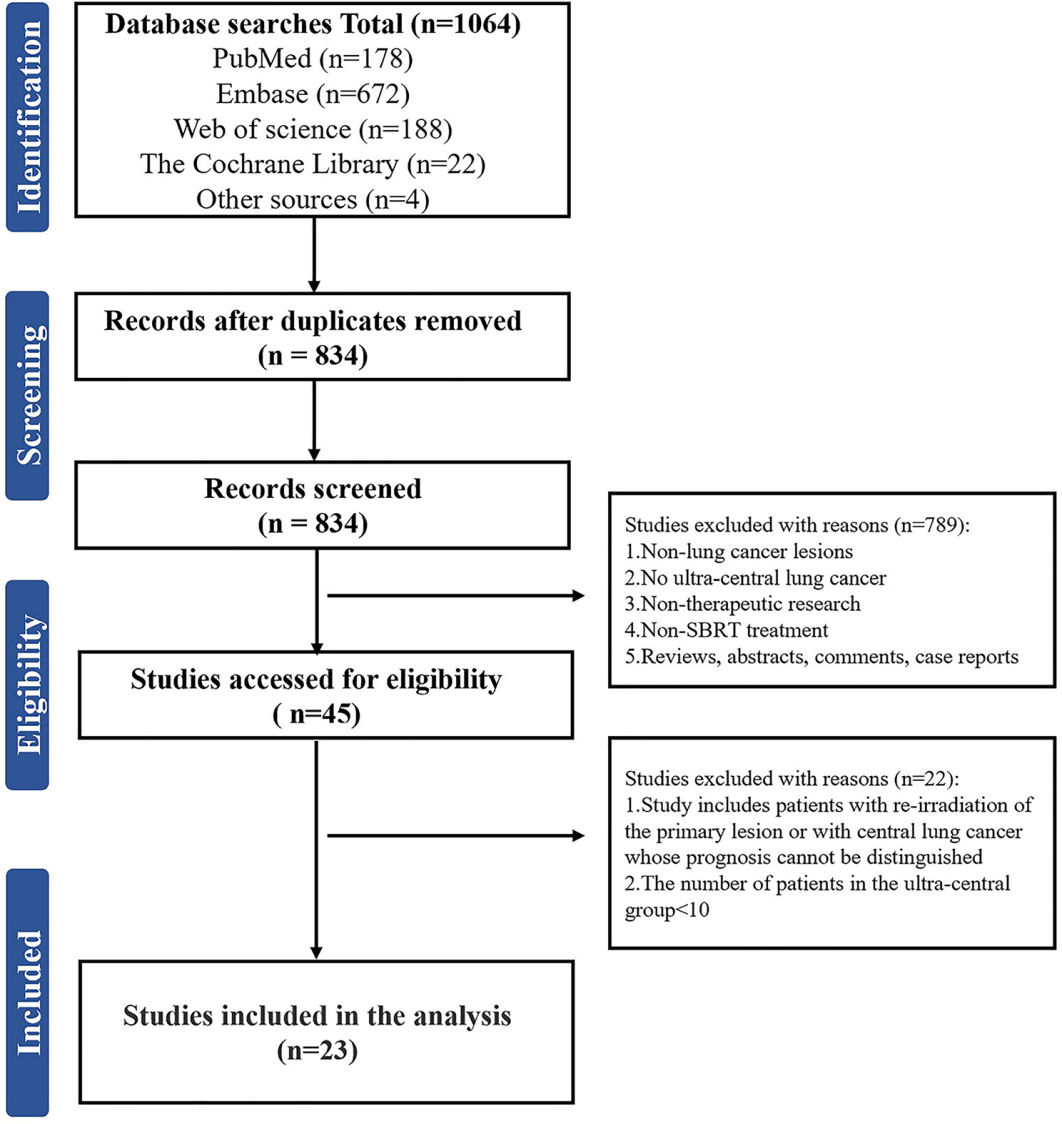
PRISMA flow diagram of study selection

The title and abstract of each study were reviewed by two reviewers, thereby creating a preliminary set of potentially relevant publications (Fig. 1). Then, two authors independently reviewed the full articles, excluded unqualified studies, and extracted data, reaching consensus on all variables.

For each study, we systematically extracted core parameters from included studies, including: first author’ s name, year of publication, country of origin, study design, sample size, gender, age, TNM stage classification, tumor histology type, definition of ultra-central lung malignancies, radiation delivery technique, planning target volume (PTV) size with range, dose per fraction, number of fractions, biological effective dose (BED₁₀), follow-up duration, 1-year and 2-year LC rates, 1-year and 2-year OS rates, and incidence of grade ≥ 3 and grade 5 side effects (including specific toxicity types and event rates). For studies that did not explicitly report the 1-year and 2-year LC rates or OS rates but provided Kaplan-Meier (K-M) curves, the relevant data points will be digitized from the K-M curves using Engauge Digitizer (version 12.1) [16, 17]. The extracted values will be clearly indicated in the summary table.

### Methodological quality assessment

The ROBINS-I tool (Risk of Bias in Non-randomized Studies of Interventions) was used to assess the quality of included studies [18].

### Statistical analyses

To provide comprehensive estimates of clinical outcomes and toxicities, we performed meta-analyses of clinical outcome proportions across the included studies. Single-arm pooled estimates for 1-year LC, 2-year LC, 1-year OS, 2-year OS, grade ≥ 3 side effect rates, and grade 5 side effect rates were derived using random-effects meta-analysis with logit transformation. Continuity correction of 0.5 was applied to studies with zero events. Study weights incorporated inverse variance weighting adjusted for within-study binomial variance and between-study heterogeneity (τ² estimated via maximum likelihood). Pooled proportions with 95% confidence intervals (CIs) were visualized through forest plots weighted by study precision, providing integrated estimates of clinical endpoints independent of dose considerations. Funnel plot and the Egger’s test were performed to describe the bias of publication, and *P* > 0.05 indicates that there was no publication bias [19, 20]. Where significant bias was detected (*P* ≤ 0.05), we implemented the trim-and-fill method to impute theoretically missing studies and recalculate the pooled effect size, providing an adjusted estimate that accounts for potential publication bias. Sensitivity analyses were performed by sequentially excluding each study to assess the robustness of the results. We performed meta-regression analyses to evaluate the associations between 1- and 2-year LCR and OS rates and the relevant predictor variables: planning target volume (PTV) and BED₁₀. The meta-regression employed univariable generalized linear mixed models (GLMM) weighted by study-specific inverse variance, incorporating continuity correction (0.5) for zero-event studies.

All statistical analyses, including meta-regression and meta-analyses, were performed in R (version 4.4.1; R Foundation for Statistical Computing) [21]. The meta-regression utilized the ‘metafor’ package (version 4.6-0), while the proportion meta-analyses were conducted using the ‘meta’ package (version 8.0–1). *P* ≤ 0.05 was considered statistically significant, all the P values were two sided.

## Results

### Study descriptions

The electronic search initially yielded 1,064 potentially relevant publications. After deduplication and screening, a total of 23 studies [6, 12, 22–42] published between 2010 and 2024, involving 1,105 unique patients, met the criteria for study inclusion (Fig. 1). Among these studies, three were prospective single-arm trials [12, 39, 41], and the other studies were retrospective studies. Median follow up ranged from 10 to 57 months for studies reporting this value. Studies were heterogeneous for the proportion of primary NSCLC (T1-4N0M0) versus metastatic/recurrent disease, with 4 studies [22, 29, 38, 39] consisting entirely of the former. The characteristics of all included studies were detailedly presented in Table 1.

### Quality assessment

The details of assessment regarding the included studies are shown in Supplementary Table 3. Notably, the majority of the studies included in this meta-analysis are small-scale, retrospective, and involve heterogeneous patient populations; therefore, the inherent limitations of the available evidence must not be overlooked.

### SBRT delivery

Radiotherapy details of all included studies are summarized in Table 2. Radiation dose and fractionations ranged from 30 to 70 Gy in 3–12 fractions. The most common dose fractionation regimen was 60 Gy/8 fractions. Notably, various cancer centers utilized distinct treatment platforms for the delivery of SBRT, with some institutions employing multiple systems, such as the CyberKnife and linear accelerators. For instance, the study conducted by Guillaume et al. [32]. utilized three distinct machines, specifically CyberKnife^®^ (Accuray, Sunnyvale), Synergy^®^ (Elekta, Stockholm, Sweden), and Versa HD^®^ (Elekta, Stockholm, Sweden), for the administration of SBRT.

**Table 2:** Treatment characteristics, dosimetric parameters, and local control outcomes of the 23 included studies

Study (Year)	PTV (cc), Median (Range)	Delivery Method	BED₁₀ (Gy)	Motion Management	Prescription (Dose/Fx, Isodose, Coverage)	Central Dose D_max_ (Gy) Bronchus / Esophagus	1-y LC (%)	2-y LC (%)	1-y OS (%)	2-y OS (%)
Unger (2010)	73 (23–324)	CyberKnife	59.5 (48–72)	Real-time tracking	30–40/5, 76% iso, ≥ 95% PTV	42^†^ / 25^†^	63	40*	54	34*
Tekatli (2016)	104.5 (17.7–508.5)	VMAT	90	4D-CT + ITV	60/12, 95% PTV, D_max_ ≤140%	69.4^‡^ / 43.6^‡^ (no G3 + group)	97*	89*	61.5	28.7
Haseltine (2016)	NA	IMRT	85.5 (85.5–100)	4D-CT + ITV	45–50/5, 100% iso, PBT D_max_ <110%	44.9–51.4^§^ / NR	NA	NA	NA	NA
Lischalk (2016)	85.8 (22.6–300.0)	CyberKnife	72 (59.5–72)	Real-time tracking	35–40/5, 75.5% iso, ≥ 95% PTV	46.7^†^ / 28.7^†^	70.1	57.4	75	40
Raman (2018)	68.5 (20.1–238.3)	IMRT/VMAT	105.0 (75.0–123.6)	4D-CT + ITV, abdominal compression	48–60/4–10, 80% iso, D99% >90% Rx	NR / NR	100	100	NA	NA
Cong (2019)	111.3 (9.8–688.9)	CyberKnife	59.5 (48–65.6)	4D-CT + ITV	30–37.5/4–6, 68–75% iso, ≥ 95% PTV	NR (29% exceeded) / NR (15% exceeded)	54.4	45.6*	76.5	38.9
Nguyen (2019)	NA	IMRT + CBCT	100 (72–105)	4D-CT + ITV, abdominal compression	40–60/5–8, 56/8 (21%), ≥ 95% PTV	55.9^‡^ / 27.0^‡^	100*	89	76*	76
Regnery (2020)	136.8 (SD ± 98.9)	3DRT/Helical Tomotherapy/ VMAT	75	4D-CT, abdominal compression	50/10, PBT D_max_ ≤44 Gy (C), D0.5 cc ≤ 52 Gy (UC)	NR (BED₃ 136 ± 16) / NR	92.1	73.1	81.2	54.9
Cooke (2020)	32.8 (8.3–106.4) (Entire cohort)	IMRT/VMAT + CBCT	105	4D-CT + ITV, CBCT	60/8, OAR constraints prioritized, PTV D_max_ 120–130%, PTV ≥ 70% Rx	43.3^‡^ / 22.3^‡^	94.1	NA	84.4	NA
Yang (2020)	36.5 (16.4–133.1)	VMAT	105	4D-CT + MIP	60/8, 95% PTV	55.1^‡^ / 19.2^¶^	92.9	92.9	87.6	76.6
Zhao (2020)	42.1 (6.6–184.8)	3DCRT/IMRT /VMAT	105	4D-CT + ITV	60/8, 90% iso, V100% >95% PTV	NR / NR	97.6*	94.5*	89.8*	79.7*
Lindberg (2021)	43.1 (9.8–180.0)	VMAT/IMRT/Static fields	95.2	4D-CT, abdominal compression	56/8, ~ 67% iso, ≥ 95% PTV, OAR inside: PTV ≥ 80% Rx	97^†^ (EQD₂) / NR	85	83	81	58
Lodeweges (2021)	55.6 (IQR 30.9–111.0)	VMAT/IMRT	90	4D-CT, ITV+3 mm	60/12, D95% ≥60 Gy, D99% ≥54 Gy, D_max_ ≤145%	48.1^‡^ (Dmean in ≥G3) / NR	98	85	77	52
Mihai (2021)	57.4 (7.7–426.6)	IMRT + motion management	105 (72–105.6)	4D-CT, gating/DIBH	40–60/4–10, homogeneous (D_max_<120%), 98% iso	52.2^‡^ (D0.1 cc) / 24.3^‡^ (D5cc)	NA	92	74.9*	55.1
Breen (2021)	56.5 (7.4–477.5)	VMAT/3DRT/DIBH	100 (75–151)	4D-CT, DIBH (10%)	50/5 (57%), 60/8 (15%), 48/4 (13%), iGTV+5 mm	PBT BED₃ >220 Gy (HR 2.19) / NR	96	84	78	57
Loi (2021)	NA	VMAT	105 (75–132)	4D-CT + ITV	50/5, 45/6, 48–60/8, 50–70/10, OAR constraints prioritized	222^†^ (BED₃) / 51^†^ (BED₃)	88	78	88	55
Guillaume (2021)	51.6 (6.6–243)	CyberKnife/VersaHD /Synergy	82 (28–105)	4DCT/3DCT, CK/VersaHD/Synergy	40–50/5–10, 80% iso, PTV = GTV/ITV+5 mm	65^†^ (EQD₂) / 22^†^ (EQD₂)	96.7	87.6	86.2	61.2
Farrugia (2021)	33.6 (23.0–52.6)	3DCRT/VMAT	100(100–132)	4D-CT, abdominal compression/gating	50–60/5, 95% PTV	52.9^‡^ / 8.95^‡^ (D5cc)	NA	NA	81*	53.3
Wang (2022)	60.2 (12.9–265.0)	CyberKnife	100.8 (95.2–111.5)	4D-CT, fiducial tracking	56/6–8, 72% iso, PTV = GTV/ITV+5 mm	56.4^‡^ / 31.1^‡^	91.5	78	94.7	75
Salvestrini (2022)	Fiducial-tracking group: Mean 53.3 (5.1–289.0) Without markers group: Mean 64.6 (10.9-231.7)	CyberKnife	92 (83–132)	4D-CT, fiducial/ITV	45–60/5–7, 60–87% iso, ≥ 95% PTV	NR (distance only) / NR	86	78	75	58
Regnery (2023)	54.7 (IQR 25.4–79.5)	MR-guided adaptive RT	75 (48–105)	SMART, DIBH, gating, daily adaptive	45–60/3–10, 95% PTV, homogeneous if needed	85^†^ (EQD₂) / NR	100	97	87	67
Giuliani (2024)	NA	SBRT+4D-CT/CBCT	105	4D-CT, motion mitigation, ITV	60/8, 95% PTV, D_max_ ≤120%	NR / NR	89.6*	89.6*	93.3*	76.5*
Lee (2024)	18.1 (3.7–359.9) (Entire cohort)	MR-guided adaptive RT	100	SMART, breath-hold, gating, daily adaptive	35–60/5, isotoxic, PTVopt, hard OAR constraints prioritized	PBT V40 100% met / Esophagus V35 100% met	94	86	72	60

Abbreviations: PTV, planning target volume; BED₁₀, biological effective dose (α/β = 10); Dmax, maximum point dose; Dmean, mean dose; D0.1 cc, D0.5 cc, D5cc, minimum dose to hottest 0.1 cc, 0.5 cc, or 5 cc; V40, V35, volume receiving ≥ 40 Gy or ≥ 35 Gy; LC, local control; OS, overall survival; NR, not reported; NA, not available; iso, isodose line; Rx, prescription dose; PBT, proximal bronchial tree; TPBT, trachea and proximal bronchial tree; ITV, internal target volume; iGTV, internal gross tumor volume; PTVopt, optimization PTV (GTV+5 mm minus abutting OARs); GTV, gross tumor volume; CTV, clinical target volume; MIP, maximum intensity projection; 4D-CT, four-dimensional computed tomography; CBCT, cone-beam CT; VMAT, volumetric modulated arc therapy; IMRT, intensity-modulated radiation therapy; 3DCRT, three-dimensional conformal radiotherapy; CK, CyberKnife; SMART, stereotactic MR-guided adaptive radiotherapy; DIBH, deep inspiration breath hold; OAR, organ at risk; C, central tumor; UC, ultracentral tumor; ULT, ultracentral lung tumor; EQD₂, equivalent dose in 2 Gy fractions (α/β = 3); BED₃, biologically effective dose (α/β = 3); HR, hazard ratio; IQR, interquartile range; SD, standard deviation. Notes: ^*^Value extracted from Kaplan-Meier curve; ^†^Mean maximum point dose; ^‡^Median (range); ^§^Range in specific patient subgroup (4 fatal toxicity cases); ^¶^Single patient value. BED₃ and EQD₂ values are indicated where reported instead of physical dose. Motion Management summarizes key respiratory motion control techniques. Prescription includes dose/fraction, fractions, isodose line, and coverage requirements. Central Dose shows maximum point dose to bronchus/PBT (left) and esophagus (right) unless specified. NR indicates not reported; NA indicates not available. Percentage values (e.g., “29% exceeded”) indicate proportion of patients exceeding dose constraints

### Local control

**Fig. 2:**
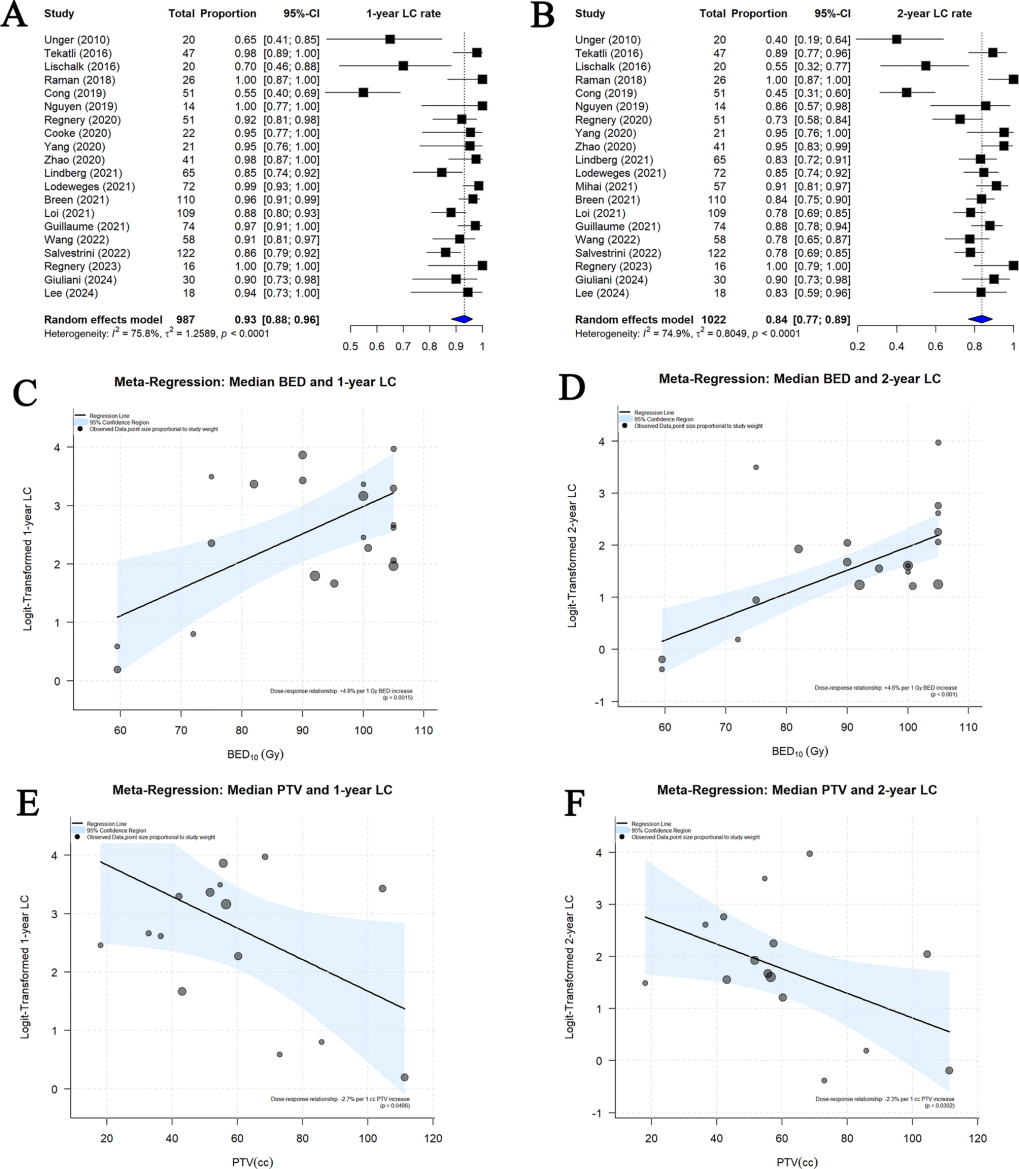
Forest plots and meta-regression analyses for local control outcomes. (**A**) Pooled 1-year local control (LC) rate. (**B**) Pooled 2-year LC rate. (**C**) Meta-regression of biologically effective dose (BED₁₀) and logit-transformed 1-year LC rate. (**D**) Meta-regression of BED₁₀ and logit-transformed 2-year LC rate. (**E**) Meta-regression of planning target volume (PTV) and logit-transformed 1-year LC rate. (**F**) Meta-regression of PTV and logit-transformed 2-year LC rate

The 1-year LC rate was reported or could be estimated from Kaplan-Meier curves in 20 studies and ranged from 54.4% to 100% (Table 2). A pooled 1-year LC rate of 93% (95% CI: 88%–96%) is shown in Fig. 2A. Despite significant heterogeneity (I² = 75.8%, *P* < 0.001), sensitivity analysis confirmed the robustness of this estimate, as the exclusion of any single study did not substantially alter the pooled result (Supplementary Fig. 4A). Meta-regression revealed a significantly positive association between BED_10_ and the 1-year LC rate (*P* = 0.001; Fig. 2C), and a significantly negative association between PTV volume and the 1-year LC rate (*P* = 0.048; Fig. 2E). Although publication bias using Egger’s test and funnel plot was detected (Supplementary Fig. 5A), trim-and-fill analysis accounting for 6 imputed studies yielded an adjusted estimate of 89.8%, indicating only a minor attenuation of the treatment effect (Supplementary Fig. 6A).

The 2-year LC rate was reported or could be estimated from Kaplan-Meier curves in 20 studies and ranged from 40% to 100% (Table 2). A pooled 2-year LC rate of 84% (95% CI: 77%–89%; I^2^ = 74.9%, *P* < 0.001) is shown in Fig. 2B, and sensitivity analysis confirmed the robustness of this estimate, demonstrating that exclusion of any single study did not substantially alter the pooled result (Supplementary Fig. 4B). Meta-regression revealed a significantly positive association between BED_10_ and the 2-year LC rate (*P* < 0.001; Fig. 2D), and a significantly negative association between PTV volume and the 2-year LC rate (*P* = 0.030; Fig. 2F). No publication bias was detected for the pooled 2-year LC rate using Egger’s test and funnel plot analysis (Supplementary Fig. 5B).

In the subset of patients with primary NSCLC (T1-4N0M0) only, the pooled 1- and 2-year LC rates were 94% (95% CI: 90%–97%; Supplementary Fig. 1A) and 83% (95% CI: 77%–87%; Supplementary Fig. 1B).

### Side effects

**Fig. 3:**
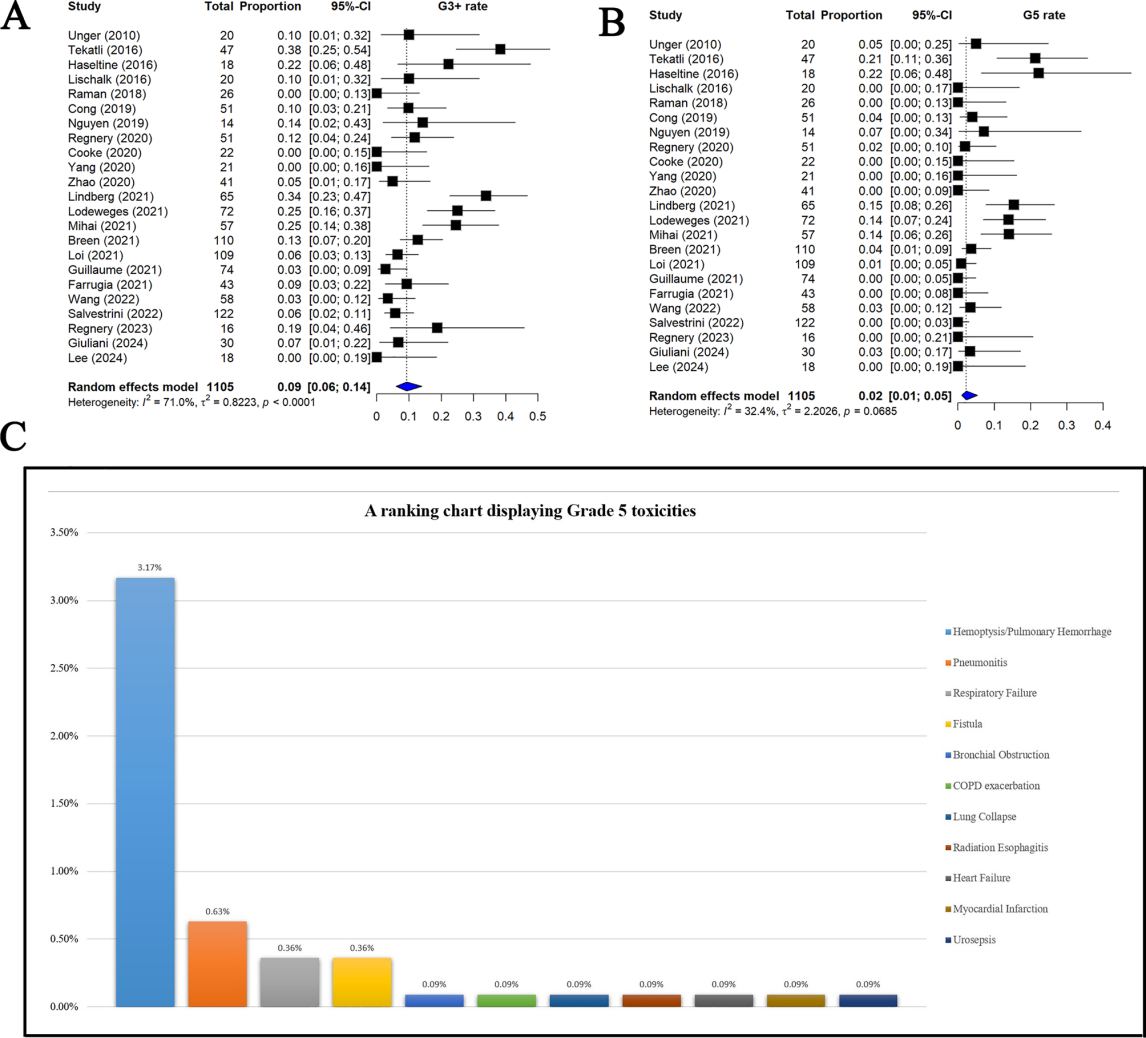
Treatment-related toxicities. (**A**) Forest plot of grade ≥ 3 toxicity incidence. (**B**) Forest plot of grade 5 (fatal) toxicity incidence. (**C**) A ranking chart displaying Grade 5 toxicities

All studies reported grade ≥ 3 side effect rates (Table 3). The pooled incidence was 9% (95% CI: 6–14%; Fig. 3A), with significant heterogeneity (I² = 71.0%, *P* < 0.001) that did not substantially affect the stability of the estimate in sensitivity analyses (Supplementary Fig. 4E). Evidence of publication bias was noted (Supplementary Fig. 5E), and trim-and-fill adjustment accounting for 6 imputed studies resulted in a 11.3% incidence (Supplementary Fig. 6B), representing a 2.3% point increase that underscores the robustness of our primary safety conclusion while acknowledging potential underreporting of adverse events.

**Table 3:** Summary of Grade 3 + and G5 toxicities of included studies

Study (Year)	Grade ≥ 3 Toxicity [ %(*n*/*N*)]	Grade 5 Toxicity [ %(*n*/*N*)]	CTCAE
Unger (2010)	10% (2/20): G3 pneumonitis (1), G5 fistula (1)	5% (1/20): G5 fatal fistula (1)	Version 3.0
Tekatli (2016)	38% (18/47),G3 radiation pneumonitis (5),G3 dyspnea or cough(3),G3 chest wall pain (2),G4 hemoptysis (1), Grade 5 Toxicity (10). Some patients had ≥ 2 types of grade 3 + toxicities	21% (10/47): G5 fatal pulmonary hemorrhage (7), G5 bronchial obstruction (1), G5 respiratory failure (2)	Version 4.0
Haseltine (2016)	24.8% (1-year cumulative incidence)	22.2% (4/18): fatal pulmonary hemorrhage (2), pneumonia (1), respiratory failure (1)	NA
Lischalk (2016)	10% (2/20): G3 pneumonitis (1), G4 atelectasis (1)	0%	Version 3.0
Raman (2018)	0%	0%	Version 3.0
Cong (2019)	9.8% (5/51): G3 pneumonitis (3), G5 heart failure (1), G5 myocardial infarction (1)	3.9% (2/51): G5 heart failure (1), G5 myocardial infarction (1)	Version 4.0
Nguyen (2019)	14.3% (2/14): G3 post-obstructive pneumonia (1), G5 respiratory failure (1)	7.1% (1/14): G5 fatal respiratory failure (1)	Version 4.03
Regnery (2020)	11.8% (6/51): G3 hemoptysis (1), G5 urosepsis (1), G3 pleural effusion (3), G3 pneumonitis (1)	2.0% (1/51): G5 urosepsis (possibly related)	Version 5.0
Cooke (2020)	0%	0%	Version 4.0
Yang (2020)	0%	0%	Version 4.0
Zhao (2020)	4.9% (2/41): dyspnea (1), hemoptysis (1)	0%	Version 5.0
Lindberg (2021)	33.8% (22patients/65):G5 bleeding (8),G5 lung infection/Pneumonitis (2),G4 pneumothorax (1),G4 lung infection/Pneumonitis (2),G4 pain (1),G4 fever (1),G4 Ventricular arrhythmia (1),G3 dyspnea (7),G3 COPD (1), G3 COPD (1),G3 lung infection/Pneumonitis (2),G3 Atrioventricular block (1),G3 Empyema (1),G3 fatigue (2),G3 pain (2),G3 gastric ulcer (1). Some patients had ≥ 2 types of grade 3 + toxicities.	15.4% (10/65): bronchopulmonary hemoptysis (8); pneumonitis (1); tracheoesophageal fistula (1)	Version 4.0
Lodeweges (2021)	Toxicity occurred during treatment: 4.2% (3/72): G3 dyspnea (1), fatigue (2); Toxicity occurred after treatment: 21% (15/72): G5 hemorrhage (10), G3 dyspnea (3), G3 pneumonitis (2), G3 fatigue (2), G5 fistula (2), G3 cough (1). Some patients had ≥ 2 types of grade 3 + toxicities	14% (10/72): G5 bronchopulmonary hemorrhage (10), including 2 cases (2.8%) caused by bronchial fistula.	Version 5.0
Mihai (2021)	Acute toxicity: 5.3% (3/57), G3 pneumonitis (2), G3 fatigue (1), G3 skin toxicity (1), one patient developed both G3 pneumonitis and G3 skin toxicity Late toxicity: 19.3% (11/57): G3 aspiration (1), G3 dyspnea (1), G3 fatigue (1), G5 hemoptysis (5), G5 pneumonia (2), G5 COPD (1)	14.0% (8/57): G5 fatal hemoptysis (5), G5 pneumonia (2), G5 COPD exacerbation (1)	Version 4.0
Breen (2021)	Acute toxicity: 5% (5/110): G3 pneumonitis (1), G5 pneumonitis (1), G3 hemoptysis (2), G3 esophagitis (1). Late toxicity: 8% (9/110): G5 pulmonary-related (4), G3 chest pain (1), G3 vertebral fracture (3), G3 esophagitis (1)	3.6% (4/110): G5 pneumonitis (1), G5 hemoptysis (1), G5 lung collapse (1), G5 fatal hemoptysis during bronchoscopy (1)	Version 5.0
Loi (2021)	6.4% (5/109): G3 pneumonitis (2), G3 hemoptysis (1), G3 bronchial stenosis (1), G5 radiation esophagitis (1)	0.9% (1/109): G5 Radiation esophagitis (1)	Version 4.0
Guillaume (2021)	2.7% (2/74): G3 pneumonitis (1), G3 oesophagitis (1)	0%	Version 4.0
Farrugia (2021)	9.3% (4/43): G3 cough (1), G3 chest wall pain (2), G3 dysphagia (1)	0.0%	Version 5.0
Wang (2022)	3.5% (2/58): G5 pneumonitis (1), G5 hemoptysis (1)	3.5% (2/58): G5 pneumonitis (1), G5 hemoptysis (1)	Version 5.0
Salvestrini (2022)	5.7% (7/122): G3 dyspnea (5), G3 pain (1), G3 fatigue (1)	0%	version 5.0
Regnery (2023)	19% (3/16): G3 bronchial bleeding (1), G4 bronchial bleeding (1), G3 esophagitis (1)	0%	Version 5.0
Giuliani (2024)	6.7% (2/30): G3 dyspnea (1), G5 pneumonia (1)	3.3% (1/30): G5 Pneumonia (1)	Version 4.0
Lee (2024)	0%	0%	Version 5.0

Abbreviations: CTCAE, Common Terminology Criteria for Adverse Events. Toxicity data presented as % (n/N), where n = number of events, N = total patients

The pooled incidence of grade 5 side effects was 2% (95% CI: 1%–5%; I^2^ = 32.4%, *P* = 0.068; Fig. 3B), and there was no significant heterogeneity for the result. Publication bias was detected for the pooled incidence of grade 5 side effects (Supplementary Fig. 5F). To address this, a trim-and-fill analysis was performed, which imputed 9 studies and yielded an adjusted incidence of 3.6%—a 1.3%-point increase from the original estimate (Supplementary Fig. 6C).

The most common grade ≥ 3 side effect was hemoptysis/pulmonary hemorrhage (41 events, Supplementary Table 4, Supplementary Fig. 3). Figure 3C displays the distribution of grade 5 side effect types in a ranking plot. Grade 5 side effects occurred in 53 events, of which 35 were hemoptysis/pulmonary hemorrhage. Supplementary Table 5 describes the studies that reported in detail the types of grade 5 toxicities, and the largest of these was the HILUS trial [12]. The key or potentially relevant risk factors related to grade 5 side effects reported in the included studies are also summarized in Supplementary Table 5.

### Overall survival

**Fig. 4:**
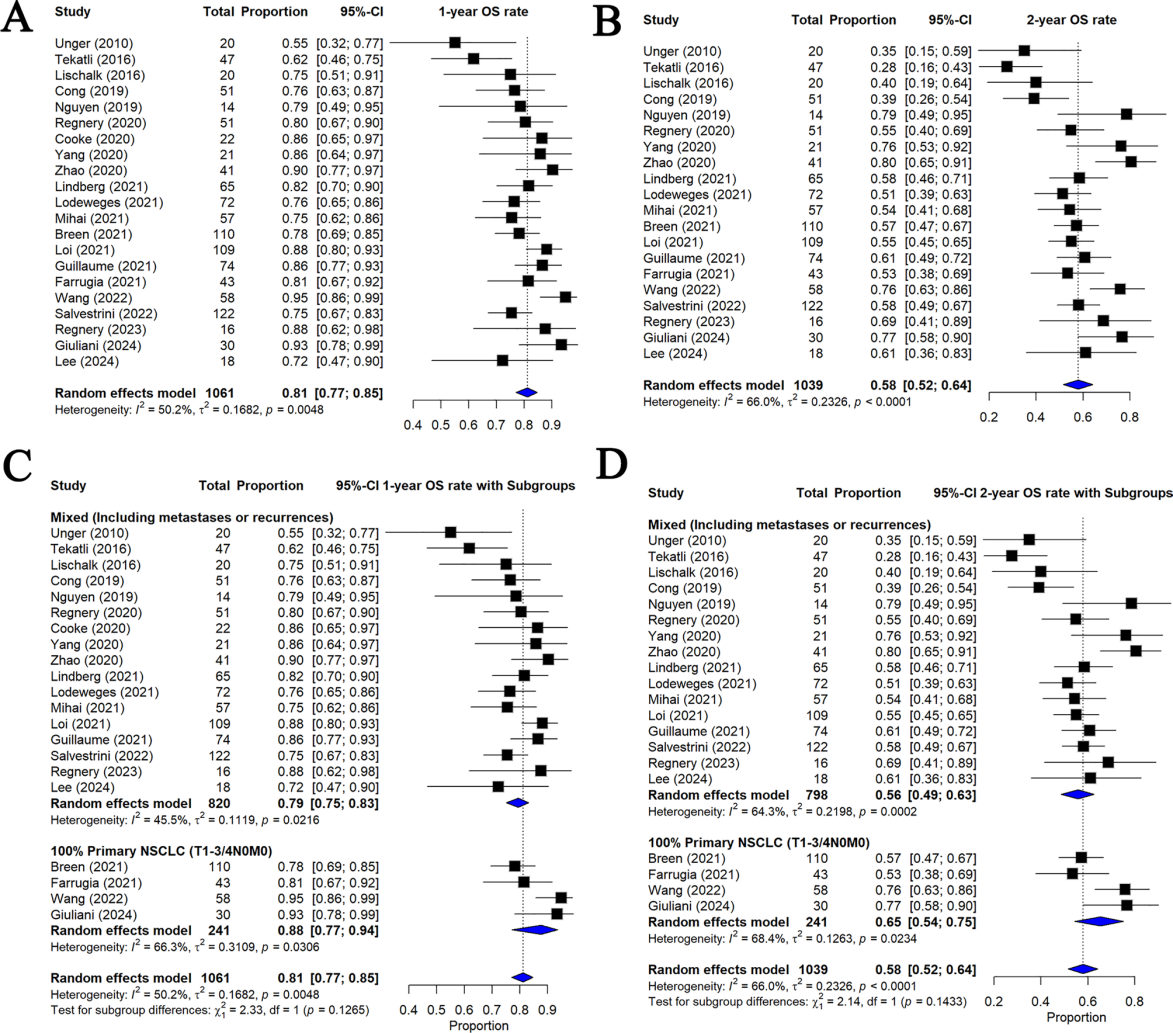
Forest plots for overall survival (OS). (**A**) Pooled 1-year OS. (**B**) Pooled 2-year OS. (**C**) Subgroup analysis of 1-year OS by tumor type. (**D**) Subgroup analysis of 2-year OS by tumor type

The 1-year OS rate was reported or could be estimated from Kaplan-Meier curves in 21 studies and ranged from 54% to 94.7% (Table 2). A pooled 1-year OS rate of 81% (95% CI: 77%–85%; I^2^ = 50.2%, *P* = 0.004) is shown in Fig. 4A. Sensitivity analysis confirmed the robustness of this estimate, as the exclusion of any single study did not substantially alter the pooled result (Supplementary Fig. 4C). Meta-regression revealed a significantly positive association between BED_10_ and the 1-year OS rate (*P* = 0.025; Supplementary Fig. 2A), and a significantly negative association between PTV and the 1-year OS rate (*P* = 0.031; Supplementary Fig. 2C). No publication bias was detected for the pooled 1-year OS rate (Supplementary Fig. 5C).

The 2-year OS rate was reported or could be estimated from Kaplan-Meier curves in 20 studies and ranged from 28.7% to 79.7% (Table 2). A pooled 2-year OS rate of 58% (95% CI: 52%–64%; I^2^ = 66.0%, *P* < 0.001) is shown in Fig. 4B. Sensitivity analysis confirmed the robustness of this estimate (Supplementary Fig. 4D). Meta-regression revealed a significantly positive association between BED_10_ and the 2-year OS rate (*P* = 0.002; Supplementary Fig. 2B), and a significantly negative association between PTV and the 2-year OS rate (*P* < 0.001; Supplementary Fig. 2D). No publication bias was detected for the pooled 2-year OS rate using Egger’s test and funnel plot analysis (Supplementary Fig. 4D).

In the subset of patients with primary NSCLC (T1-4N0M0) only, the pooled 1- and 2-year OS rates were 88% (95% CI: 77%–94%; Fig. 4C) and 65% (95% CI: 54%–75%; Fig. 4D).

## Discussion

Over the past decade, there has been a growing adoption of SBRT for the management of UC lung malignancies. Nevertheless, concerns persist regarding its efficacy and tolerability. In this study, we conducted a pooled analysis of the existing data on the application of SBRT for UC lung tumors, and performed a comprehensive analysis of its efficacy and side effects within this specific clinical setting. The key findings of this study are summarized as follows. (1) A significant dose–response relationship for local tumor control in SBRT was both observed and quantified. (2) PTV volume is negatively associated with 1- and 2-year LC rates. (3) The pooled 1-year and 2-year LCR reached 93% and 84%, respectively. (4) Severe side effects were not negligible, with a pooled grade 5 side effects rate of 2%.

Although previous studies have suggested the possibility of a SBRT dose–response relationship, this relationship remains inconclusive and unquantified. The landmark HypoFXSRT study (2007) demonstrated that a BED_10_ of > 100 Gy resulted in an LC rate of 91.6% for stage I NSCLC, compared to 57.1% for BED_10_ below 100 Gy [43]. Notably, in the HypoFXSRT study, all patients had small lung tumors (T1-2N0M0), and it is questionable whether a BED_10_ of > 100 Gy is similarly effective for larger lesions (e.g., T3N0M0). In a systematic review and meta-analysis by Senthi et al. (2013), it was found that the LC rate was ≥ 85% when the BED_10_ was ≥ 100 Gy [9]. In a systematic review and meta-analysis by Rim et al. (2020) [44], they found that a BED_10_ of ≥ 85 Gy resulted in a significantly higher 1-year LC rate compared with a BED_10_ below 85 Gy (94.4% vs. 59.3%, *P* < 0.001). Thus, they recommended a BED_10_ dose of at least 85 Gy for SBRT of UC lung tumors [44]. A recent systematic review and meta-analysis by Yan et al. (2023) suggested that there is approximately a 90% local control probability at a BED_10_ of 100 Gy for UC lung tumors of any histology [14]. Consistent with previous studies, we demonstrated a dose–response relationship in which increasing BED_10_ was significantly correlated with higher local control probability using meta-regression analysis. Each 1 Gy increase in BED_10_ was associated with a 4.8% and 4.6% increase in 1- and 2-year LC rates (Fig. 2C and D, respectively), underscoring the importance of optimizing radiation dose within clinically acceptable limits to improve LCR of UC lung lesions receiving SBRT. Besides, the positive association between higher BED₁₀ and improved 1- and 2-year OS rates may suggest that effective local control of lesions contributes to patient survival. However, this observation should be interpreted with caution due to the substantial impact of extrathoracic disease status on OS. Moreover, the observed positive association between BED₁₀ and OS may be confounded by underlying disease features. The disease characteristics of primary UC lung cancer, especially in early-stage cases, differ from those of metastatic or recurrent UC lung malignancies. In clinical practice, a palliative SBRT regimen with a lower BED_10_ is more likely to be applied to metastatic or recurrent UC lung malignancies. Notably, although the included studies in this review only covered UC lung malignancies receiving initial SBRT, a subgroup analysis distinguishing between primary and metastatic or recurrent UC lung malignancies could not be conducted due to the lack of available information for such stratification. Therefore, although a significantly positive correlation between BED₁₀ and the 1 - and 2 - year OS rates was shown by meta-regression analysis, this should not be regarded as evidence of a dose–survival effect relationship. Thus, it is necessary to emphasize that the analysis of BED₁₀ and OS was restricted to exploratory purposes.

In the present study, in line with the results of previous studies [5, 45, 46], tumor volume was found to be negatively correlated with local tumor control and OS rates. These results were not difficult to understand because larger tumors are associated with more pronounced intratumoral hypoxia, which in turn contributes to radioresistance [47, 48], immune suppression [47], and increased metastatic potential [49]. Besides, a larger tumor volume required a larger irradiation field to maintain adequate tumor coverage, resulting in greater negative effects on blood lymphocytes and more severe radiation-related side effects, given that UC lesions are located closer to the heart and the thoracic great blood vessels [5]. However, the negative correlation between tumor volume and local tumor control presents a significant challenge in the treatment of large UC tumors. Timmerman et al. [8]. demonstrated that the size of the gross tumor volume (GTV) was a significant predictor of grade 3 to 5 SBRT-related side effects, and tumors with GTV volume of more than 10 mL had an 8-fold risk of high-grade side effects compared with smaller tumors (*P* = 0.017). As such, the negative correlation between PTV and both LCR and OS further emphasizes the importance of active monitoring and timely intervention, with SBRT potentially prioritized for small-volume and early-detected UC lung malignancies due to its more favorable benefit-risk profile. Notably, emerging planning and delivery technologies may help reduce the risk of SBRT-related side effects for large-volume UC tumors. For example, the use of stereotactic MR-guided adaptive radiation therapy (SMART) can improve daily setup accuracy and both tumor and normal tissue visualization, allowing for smaller margins and reducing the risks. A recent study by Regnery et al. compared the treatment outcomes of using SMART to treat ultra-central tumors (UCT) and non-UCT, although UCTs were larger (median PTV: UCT 54.7 cm³, non-UCT 19.2 cm³). UCTs and non-UCT showed similar OS rates (2-years: UCT 67%, non-UCT 60%, *P* = 0.7) and progression-free survival (PFS) rates (2-years: UCT 37%, non-UCT 34%, *P* = 0.73) [41].

In this study, the pooled incidence of grade ≥ 3 side effects of 9% is consistent with prior reports [44], and reflects the inherent challenge of delivering ablative doses to UC lung lesions. However, the relatively low grade 5 side effect incidence (2%) suggests that the majority of treatment-related adverse effects are non-fatal. As fatal complications are the most important concern when applying SBRT for UC tumors, we qualitatively analyzed the reported toxicities in addition to the pooled analyses. Moreover, by evaluating information provided in individual studies on patients who experienced grade 5 side effects, we identified potential high-risk indicators for fatal side effects (e.g., endobronchial tumor, anticoagulant use; Supplementary Table 5).

To the best of our knowledge, this study represents the most comprehensive analysis of SBRT for UC lung malignancies. Nevertheless, our study has some limitations. Firstly, the analysis is based on published data rather than individual patient-level data, which may introduce potential uncertainties in the interpretation of side effects and restrict the scope of statistical testing. Secondly, the applicability of the LQ model is controversially discussed for the calculation of BED in high-dose per-fraction SBRT. However, previous studies demonstrated that local tumor control in fractionated SBRT for lung tumors is well modeled using the classical LQ formula [50, 51]. Thirdly, although we extracted detailed prescription and central dose parameters whenever they were available (Table 2), comprehensive dosimetric data could not be obtained from all the included studies. Additionally, there was substantial heterogeneity in the reporting standards of the already-extracted data. Last but not least, the majority of the studies included in this review are retrospective. Selection bias and potential confounding factors may influence the results. However, we employed random-effects models, conducted meta-regression, and performed sensitivity analyses to ensure the reliability of our results.

## Conclusions

In conclusion, SBRT for UC lung tumors demonstrates favorable LC rates, although it carries the risk of severe side effects. A significant dose–response relationship for tumor control probability (TCP) was identified. The identified causes of fatal toxicities should be avoided in clinical practice as much as possible. Further prospective research is warranted to verify our conclusions.

## Supplementary Information

Below is the link to the electronic supplementary material.

Supplementary Material 1: Supplementary Fig. 1. (A) Subgroup analysis of 1-year LC by tumor type. (B) Subgroup analysis of 2-year LC by disease status. Supplementary Fig. 2. (A) Meta-regression of BED₁₀ and logit-transformed 1-year OS rate. (B) Meta-regression of BED₁₀ and logit-transformed 2-year OS rate. (C) Meta-regression of PTV and logit-transformed 1-year OS rate. (D) Meta-regression of PTV and logit-transformed 2-year OS rate. Supplementary Fig. 3. Distribution of Grade ≥ 3 treatment-related toxicities. Supplementary Fig. 4. Sensitivity analyses of pooled outcomes using the leave-one-out method. Leave-one-out sensitivity analyses were performed to assess the robustness of the pooled estimates by sequentially excluding each individual study. (A). 1-year LC rate. (B). 2-year LC rate. (C). 1-year OS rate. (D). 2-year OS rate. (E). Grade ≥ 3 toxicity rate. (F). Grade 5 toxicity rate. Supplementary Fig. 5. Publication bias assessment for key outcomes using funnel plots and Egger’s test. (A). Funnel plot plots and Egger’s test for the pooled 1-year LC rate. (B). Funnel plot plots and Egger’s test for the pooled 2-year LC rate. (C). Funnel plot plots and Egger’s test for the pooled 1-year OS rate. (D). Funnel plot plots and Egger’s test for the pooled 2-year OS rate. (E). Funnel plots and Egger’s test for the pooled Grade ≥ 3 toxicity rate. (F). Funnel plots and Egger’s test for the pooled Grade 5 toxicity rate. Supplementary Fig. 6. Funnel plots with trim-and-fill adjustment for publication bias. (A) Funnel plot for the 1-year local control (LC) rate. (B) Funnel plot for the grade ≥ 3 (G3+) toxicity rate. (C) Funnel plot for the grade 5 (G5) toxicity rate.

## Data Availability

All the data are available from the corresponding author upon reasonable request.
