# Supplementary material for: Stereotactic body radiation therapy for ultra-central lung malignancies: an updated systematic review and meta-analysis
Source: Radiat Oncol. 2026 May 11;21:89. doi: 10.1186/s13014-026-02854-5 (PMC13277070; doi:10.1186/s13014-026-02854-5)
Supplement: Supplementary file 1 — Supplementary Material 1: Supplementary Fig. 1. (A) Subgroup analysis of 1-year LC by tumor type. (B) Subgroup analysis of 2-year LC by disease status. Supplementary Fig. 2. (A) Meta-regression of BED₁₀ and logit-transformed 1-year OS rate. (B) Meta-regression of BED₁₀ and logit-transformed 2-year OS rate. (C) Meta-regression of PTV and logit-transformed 1-year OS rate. (D) Meta-regression of PTV and logit-transformed 2-year OS rate. Supplementary Fig. 3. Distribution of Grade ≥ 3 treatment-related toxicities. Supplementary Fig. 4. Sensitivity analyses of pooled outcomes using the leave-one-out method. Leave-one-out sensitivity analyses were performed to assess the robustness of the pooled estimates by sequentially excluding each individual study. (A). 1-year LC rate. (B). 2-year LC rate. (C). 1-year OS rate. (D). 2-year OS rate. (E). Grade ≥ 3 toxicity rate. (F). Grade 5 toxicity rate. Supplementary Fig. 5. Publication bias assessment for key outcomes using funnel plots and Egger’s test. (A). Funnel plot plots and Egger’s test for the pooled 1-year LC rate. (B). Funnel plot plots and Egger’s test for the pooled 2-year LC rate. (C). Funnel plot plots and Egger’s test for the pooled 1-year OS rate. (D). Funnel plot plots and Egger’s test for the pooled 2-year OS rate. (E). Funnel plots and Egger’s test for the pooled Grade ≥ 3 toxicity rate. (F). Funnel plots and Egger’s test for the pooled Grade 5 toxicity rate. Supplementary Fig. 6. Funnel plots with trim-and-fill adjustment for publication bias. (A) Funnel plot for the 1-year local control (LC) rate. (B) Funnel plot for the grade ≥ 3 (G3+) toxicity rate. (C) Funnel plot for the grade 5 (G5) toxicity rate. [file 13014_2026_2854_MOESM1_ESM.pdf]

Contents

Supplementary Table 1. PICO inclusion criteria……………………………………………………….2

Supplementary Table 2. Queries used for systematic search…………………………………………..3

Supplementary Table 3. Quality assessment according to ROBINS-I…………………………………5

Supplementary Table 4. Summary of treatment-related Grade ≥3 toxicities reported………………...6

Supplementary Table 5. Grade 5 toxicity types and risk factors………………………………………7

Supplementary Figure 1. Subgroup analysis of 1/2-year LC by tumor type…………………………..8

Supplementary Figure 2. Meta-regression of BED_{10}/PTV and logit-transformed 1/2-year OS rate….9

Supplementary Figure 3. Distribution of Grade ≥3 treatment-related toxicities……………………...10

Supplementary Figure 4. Sensitivity analyses of pooled outcomes…………………………………..11

Supplementary Figure 5. Publication bias assessment using funnel plots and Egger's……………... 12

Supplementary Figure 6. Funnel plots with trim-and-fill adjustment for publication bias……..........13

PRISMA checklist……………………………………………………………………………..14

Supplementary Table 1. PICO inclusion criteria.

**P** (Population)	Adult patients with ultra-central (UC) lung tumors, defined as primary or metastatic lesions where the planning target volume (PTV) is abutting or overlapping critical mediastinal structures (e.g., proximal bronchial tree, trachea, esophagus, great vessels).
**I** (Intervention)	Stereotactic body radiotherapy (SBRT) / Stereotactic Ablative Radiotherapy (SABR):<br>Single fraction dose≥5Gy<br>Techniques include: VMAT, IMRT, CyberKnife, MRI-guided adaptive radiotherapy, etc.
**C** (Control)	None
**O**(Outcomes)	Primary outcomes:<br>1, 2-year local control rate (LC)<br>Secondary Outcomes:<br>1, 2-year overall survival (OS)<br>Toxicity: Incidence of Grade ≥3 adverse events, including hemorrhage, pneumonia, esophagitis, and cardiotoxicity.

Abbreviation: PICO: Population, Intervention, Control, Outcome.

Supplementary Table 2. Queries used for systematic search

**Pubmed (n=178)**		
Details of the literature search process		
#1	((("Lung Neoplasms"[Mesh] OR "Lung Cancer"[tiab] OR "Pulmonary Neoplasms"[tiab]) AND ("Mediastinum"[Mesh] OR "Proximal Bronchial Tree"[tiab] OR "PBT"[tiab] OR "Carina"[tiab] OR "Esophagus"[tiab] OR "Great Vessels"[tiab] OR "Aorta"[tiab] OR "Pulmonary Artery"[tiab])) OR ("Ultra-central"[tiab] OR "Ultracentral"[tiab] OR "Centrally Located"[tiab] OR "Perihilar"[tiab]))	13,258
#2	((Stereotactic Body Radiotherapy[Mesh] OR "Stereotactic Ablative Radiotherapy"[tiab] OR "SABR"[tiab] OR "SBRT"[tiab]) AND (Dose Fractionation[Mesh] OR "Radiotherapy Dosage"[Mesh] OR "Dose"[tiab] OR "Fractionation"[tiab] OR "Fractionated"[tiab] OR "Fractions"[tiab] OR "Hypofractionation"[tiab]))	13,337
#3	"Local Control"[tiab] OR "Tumor Control"[tiab] OR "LC"[tiab] OR "Recurrence"[Mesh] OR "Treatment Outcome"[Mesh] OR "Survival Rate"[Mesh] OR "1-year survival"[tiab] OR "2-year survival"[tiab] OR "Outcome Assessment"[tiab]	1,821,876
#4	#1 AND #2 AND #3	178
**Embase (n=671)**		
Details of the literature search process		
#1	('lung tumor'/exp OR 'lung tumor' OR 'lung cancer':ti,ab OR 'pulmonary neoplasm':ti,ab) AND ('mediastinum'/exp OR 'mediastinum' OR 'trachea carina' OR 'esophagus'/exp OR 'esophagus' OR 'aorta'/exp OR 'aorta' OR 'pulmonary artery'/exp OR 'pulmonary artery' OR 'large blood vessel'/exp OR 'large blood vessel' OR 'proximal bronchial tree':ti,ab OR 'pbt':ti,ab OR 'carina':ti,ab OR 'esophagus':ti,ab OR 'great vessels':ti,ab OR 'aorta':ti,ab OR 'pulmonary artery':ti,ab) OR 'ultra central':ti,ab OR 'ultracentral':ti,ab OR 'centrally located':ti,ab OR 'perihilar':ti,ab	56298
#2	('stereotactic body radiation therapy'/exp OR 'stereotactic ablative radiotherapy':ti,ab OR 'sabr':ti,ab OR 'sbrt':ti,ab) AND ('dose fractionation'/exp OR 'radiation dose'/de OR 'dose':ti,ab OR 'fractionation':ti,ab OR 'fractionated':ti,ab OR 'fractions':ti,ab OR 'hypofractionation':ti,ab)	19496
#3	'local control':ti,ab OR 'tumor control':ti,ab OR 'lc':ti,ab OR 'tumor recurrence'/exp OR 'treatment outcome'/de OR 'survival rate'/de OR '1 year survival':ti,ab OR '2 year survival':ti,ab OR 'outcome assessment':ti,ab	1673644
#4	#1 AND #2 AND #3	672
**The Cochrane Library (n=21)**		
Details of the literature search process		
#1	("Lung Neoplasms" OR "Lung Cancer" OR "Pulmonary Neoplasms" OR "Lung Tumor" OR "Lung Tumour" OR "Lung Carcinoma" OR "Pulmonary Tumor" OR "Pulmonary Tumour" OR "Pulmonary Carcinoma" OR "Non-small Cell Lung Cancer" OR "NSCLC"):ti,ab,kw	29867
#2	("Mediastinum" OR "Proximal Bronchial Tree" OR "PBT" OR "Carina" OR "Esophagus" OR "Great Vessels" OR "Aorta" OR "Pulmonary Artery"):ti,ab,kw	18141
#3	("Ultra-central" OR "Ultracentral" OR "Centrally Located" OR "Perihilar"):ti,ab,kw	262
#4	#2 OR #3	18369
#5	#1 AND #4	815
#6	("Stereotactic Body Radiotherapy" OR "Stereotactic Ablative Radiotherapy" OR "SABR" OR "SBRT" OR "Stereotactic Radiation"):ti,ab,kw	1670

#7	("Radiotherapy Dosage" OR "Dose Fractionation" OR "Fractionation" OR "Fractionate d" OR "high dose" OR "dose escalation"):ti,ab,kw	45087
#8	#6 AND #7	861
#9	("Local Control" OR "Tumor Control" OR "LC rate" OR "local recurrence" OR "local f ailure" OR "disease control"):ti,ab,kw	17249
#10	("1-year" OR "2-year" OR "12-month" OR "24-month" OR "one-year" OR "two-year "):ti,ab,kw	129722
#11	#9 OR #10	144850
#12	#5 AND #8 AND #11	22
**Web of science (n=188)**		
Details of the literature search process		
#1	((TS=("Lung Neoplasm*" OR "Lung Cancer" OR "Pulmonary Neoplasm*") AND TS= ("Mediastinum" OR "Proximal Bronchial Tree" OR "PBT" OR "Carina" OR "Esophag us" OR "Great Vessels" OR "Aorta" OR "Pulmonary Artery")) OR TS= ("Ultra-central " OR "Ultracentral" OR "Centrally Located" OR "Perihilar"))	9080
#2	(TS=("Stereotactic Body Radiotherapy" OR "Stereotactic Ablative Radiotherapy" OR " SABR" OR "SBRT")) AND (TS=("Dose Fractionation" OR "Radiotherapy Dosage" O R "Dose*" OR "Fractionation" OR "Fractionated" OR "Fraction*" OR "Hypofractiona tion"))	8732
#3	TS=("Local Control" OR "Tumor Control" OR "LC" OR "Recurrence" OR "Treatment Outcome" OR "Survival Rate" OR "1-year survival" OR "2-year survival" OR "Outco me Assessment")	682741
#4	#1 AND #2 AND #3	188

Supplementary Table 3. Quality assessment of the included studies according to ROBINS-I tool of Cochrane library. (+): low risk; (++): moderate risk.

Study	Bias due to confounding	Bias in selection of participants into the study	Bias in classification of interventions	Bias due to deviations from intended interventions	Bias due to missing data	Bias in measurement of outcomes	Bias in selection of the reported result
Unger (2010)	(++)	(++)	(+)	(+)	(++)	(++)	(++)
Tekatli (2016)	(++)	(++)	(+)	(+)	(++)	(++)	(+)
Haseltine (2016)	(++)	(++)	(+)	(+)	(++)	(++)	(++)
Lischalk (2016)	(++)	(++)	(+)	(+)	(++)	(++)	(+)
Raman (2018)	(++)	(++)	(+)	(+)	(++)	(++)	(+)
Cong (2019)	(++)	(++)	(+)	(+)	(++)	(++)	(++)
Nguyen (2019)	(++)	(++)	(+)	(++)	(++)	(++)	(+)
Regnery (2020)	(++)	(++)	(+)	(+)	(++)	(++)	(+)
Cooke (2020)	(++)	(++)	(+)	(+)	(++)	(++)	(+)
Yang (2020)	(++)	(++)	(+)	(+)	(++)	(+)	(+)
Zhao (2020)	(++)	(+)	(+)	(+)	(+)	(+)	(+)
Lindberg (2021)	(+)	(+)	(+)	(+)	(++)	(+)	(++)
Lodeweges (2021)	(++)	(++)	(+)	(++)	(++)	(++)	(++)
Mihai (2021)	(++)	(++)	(+)	(++)	(++)	(++)	(++)
Breen (2021)	(++)	(++)	(+)	(+)	(++)	(+)	(+)
Loi (2021)	(++)	(++)	(+)	(+)	(++)	(+)	(+)
Guillaume (2021)	(++)	(++)	(+)	(+)	(++)	(+)	(+)
Farrugia (2021)	(++)	(++)	(+)	(+)	(++)	(++)	(+)
Wang (2022)	(++)	(++)	(+)	(+)	(++)	(++)	(+)
Salvestrini (2022)	(++)	(++)	(+)	(+)	(++)	(++)	(+)
Regnery (2023)	(++)	(+)	(+)	(+)	(+)	(+)	(+)
Giuliani (2024)	(++)	(+)	(+)	(+)	(+)	(+)	(+)
Lee (2024)	(++)	(++)	(+)	(+)	(++)	(+)	(++)

Supplementary Table 4. Summary of treatment-related Grade ≥3 toxicities reported in the included studies

Organ system/ Toxicity type	≥3 Toxicities	Grade	Number of occurrences (n)	Frequency (%) †
**Respiratory system**	Hemoptysis/Pulmonary Hemorrhage	G5	35	3.17%
	Hemoptysis/Pulmonary Hemorrhage	G4	1	0.09%
	Hemoptysis/Pulmonary Hemorrhage	G3	5	0.45%
	Pneumonitis	G5	7	0.06%
	Pneumonitis	G4	1	0.09%
	Pneumonitis	G3	20	1.81%
	Respiratory Failure	G5	4	0.36%
	Bronchial Obstruction	G5	1	0.09%
	COPD exacerbation	G5	1	0.09%
	Lung Collapse	G5	1	0.09%
	Atelectasis	G4	1	0.09%
	Pneumothorax	G4	1	0.09%
	Dyspnea	G3	21	1.90%
	Cough	G3	5	0.45%
	Pleural Effusion	G3	3	0.27%
	COPD	G3	2	0.18%
	Bronchial stenosis	G3	1	0.09%
	Empyema	G3	1	0.09%
**Digestive system**	Fistula	G5	4	0.36%
	Esophagitis	G5	1	0.09%
	Esophagitis	G3	4	0.36%
	Gastric Ulcer	G3	1	0.09%
	Dysphagia	G3	1	0.09%
**Cardiovascular system**	Heart Failure	G5	1	0.09%
	Myocardial Infarction	G5	1	0.09%
	Ventricular Arrhythmia	G4	1	0.09%
	Atrioventricular Block	G3	1	0.09%
**Others**	Urosepsis	G5	1	0.09%
	Fever	G4	1	0.09%
	Pain (Chest Wall/General)	G3	9	0.81%
	Fatigue	G3	9	0.81%
	Vertebral Fracture	G3	3	0.27%
	Skin Toxicity	G3	1	0.09%
	Aspiration	G3	1	0.09%

Note: This table summarizes all toxicity events of grade ≥ 3 clearly listed in the original table. Since some patients experienced multiple toxicities, the total number of events exceeded the total number of patients. †Frequency (%) was calculated based on total patient number from all studies (N =1105).

Supplementary Table 5. Grade 5 toxicity types and risk factors.

Toxicity Type	Study (No. patient); Time to Event (m)	Relevant risk factors
Pulmonary Hemorrhage/ Hemoptysis	Tekatli(7); Range:5.2-18.2m	Anticoagulant use (5/7,71%)<br>Endobronchial lesion (3/7,43%)<br>Interstitial changes (3/7,43%)
	Haseltine (2); 9/10m	Anti-VEGF Therapy 100% (2/2)
	Lindberg (8); Median 15m (Range:2-22)	$D_{0.2}$cc was the strongest predictor<br>D_{max}>100 Gy (EQD_2) was observed in 7 of 8 patients.
	Lodeweges(10); Median 11m (Range:8-21)	PTV overlap main bronchus (10/10,100%)<br>Higher mean dose (D_{mean}) of the main bronchus.
	Mihai (5); Median (all events): 12m	Airway BED_3 (4 cm^3) >147.4 Gy
	Breen (2); 11/24m	Bronchial wall invasion<br>Airway Friability
	Wang (1);31m	None
Pneumonia	Haseltine (1); 8m	Abutting the PBT
	Lindberg (1); NA	NA
	Mihai (2); 7.4/11.6m	Unclear if related to SABR
	Breen (1); 6m	Pulmonary insufficiency
	Wang (1); 24m	Large PTV
	Giuliani (1); NA	Interstitial lung disease
Fistula	Unger (1); 7m	Endobronchial disease<br>Large GTV<br>High D_{max} of the main bronchus (49 Gy)
	Lindberg (1); 9m	Esophageal invasion into the PTV<br>Protocol-violating high dose (80 Gy) to the esophagus
	Lodeweges (2); NA	Fistula location in the high-dose radiation area<br>Ulceration and necrosis of the bronchus
Respiratory Failure	Haseltine (1); 7m	Tumor abutting the PBT<br>Significant clinical suspicion that SBRT was a contributing factor to the respiratory complication
	Tekatli (2); 5.6/7.7m	Multifactorial respiratory failure
	Nguyen (1); NA	High radiation dose to the PBT
Other	Urosepsis (Regnery (2020),1); NA	Possibly SBRT-related death
	Esophagitis (Loi,1); NA	Massive mediastinal recurrence<br>Radiation injury, suggested by an ulcerated and hemorrhagic mucosal lining
	Bronchial obstruction (Tekatli,1); 11.3m	COPD
	COPD exacerbation (Mihai,1); 66m	Pre-existing COPD
	Lung collapse (Breen,1); 7m	Prior contralateral pneumonectomy
	Heart failure (Cong,1); 4m	Pre-existing heart disease<br>High radiation dose to the heart
	Myocardial infarction (Cong,1);11m	Pre-existing heart disease<br>Radiation dose to the heart

Abbreviation: COPD, Chronic obstructive pulmonary disease; m, month; $D_{0.2}$cc, minimum dose to the “hottest” 0.2 cc, EQD_2, Equivalent dose in 2 Gy per fraction; PTV, Planning target volume; GTV, Gross tumor volume; VEGF, Vascular endothelial growth factor; SABR, Stereotactic ablative body radiotherapy; SBRT, Stereotactic body radiation therapy; PBT, Proximal bronchial tree.

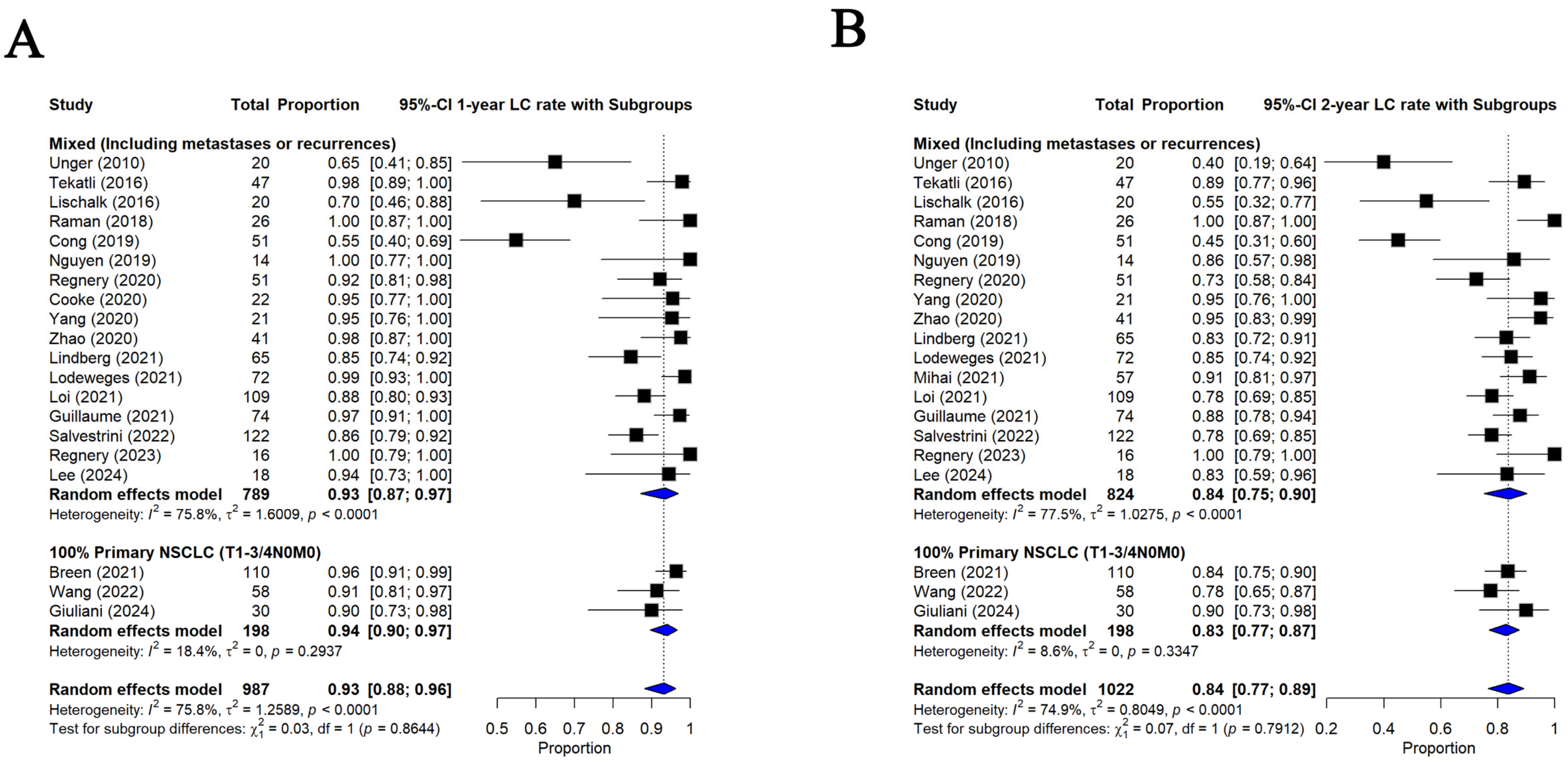

Supplementary Figure 1. (A) Subgroup analysis of 1-year LC by tumor type. (B) Subgroup analysis of 2-year LC by disease status.

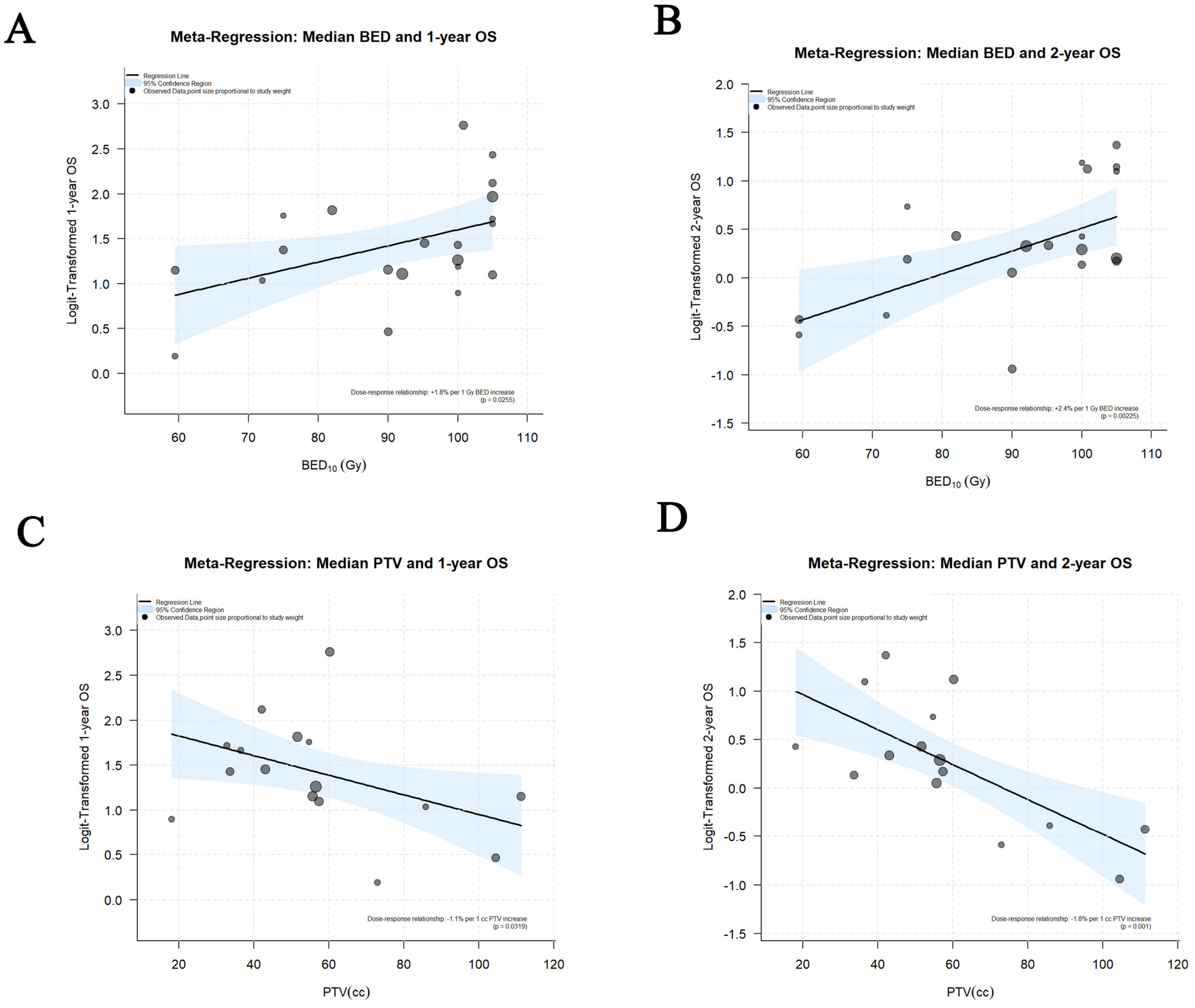

Supplementary Figure 2. **(A)** Meta-regression of BED_{10} and logit-transformed 1-year OS rate. **(B)** Meta-regression of BED_{10} and logit-transformed 2-year OS rate. **(C)** Meta-regression of PTV and logit-transformed 1-year OS rate. **(D)** Meta-regression of PTV and logit-transformed 2-year OS rate.

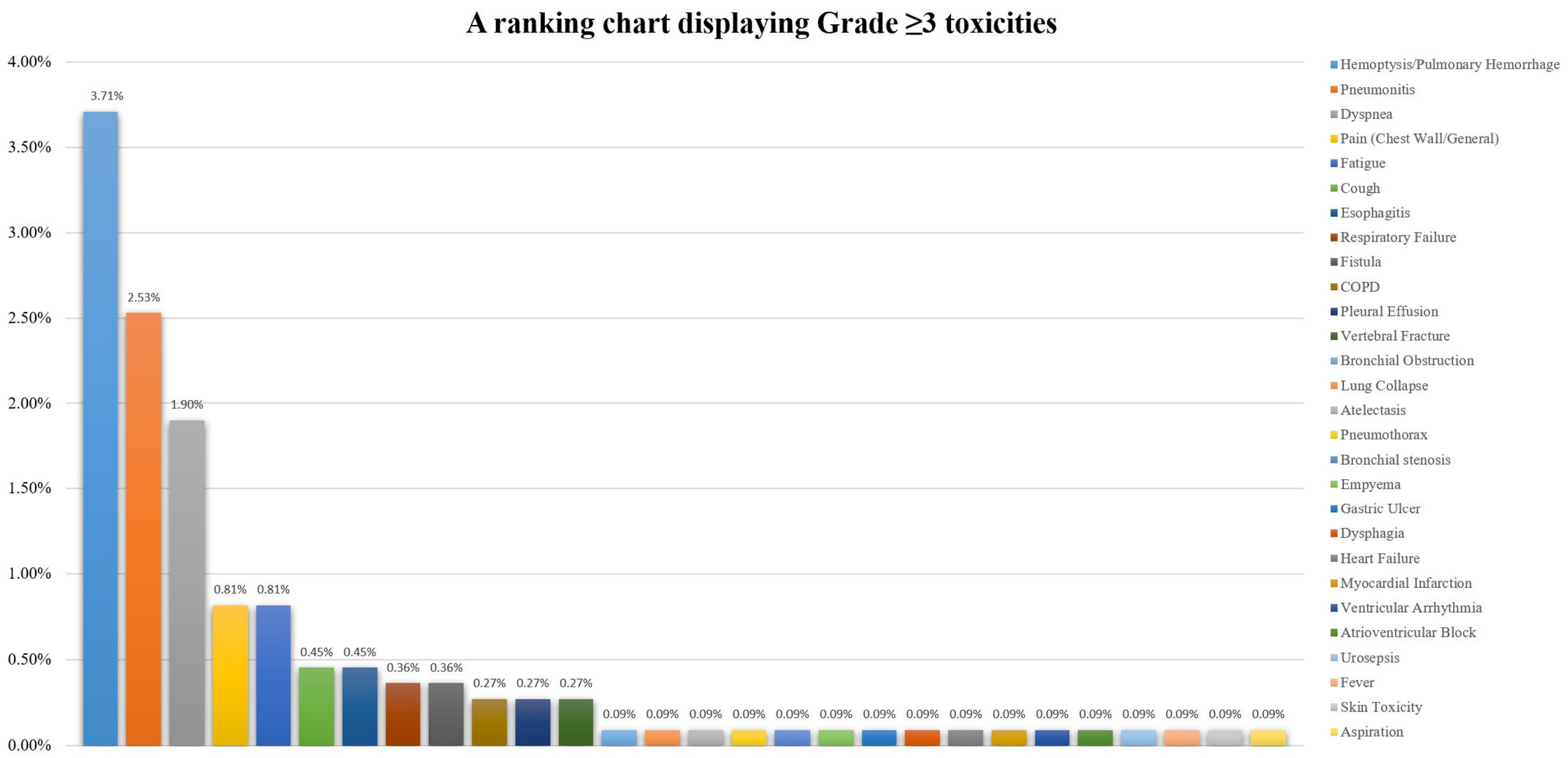

Supplementary Figure 3. Distribution of Grade ≥3 treatment-related toxicities.

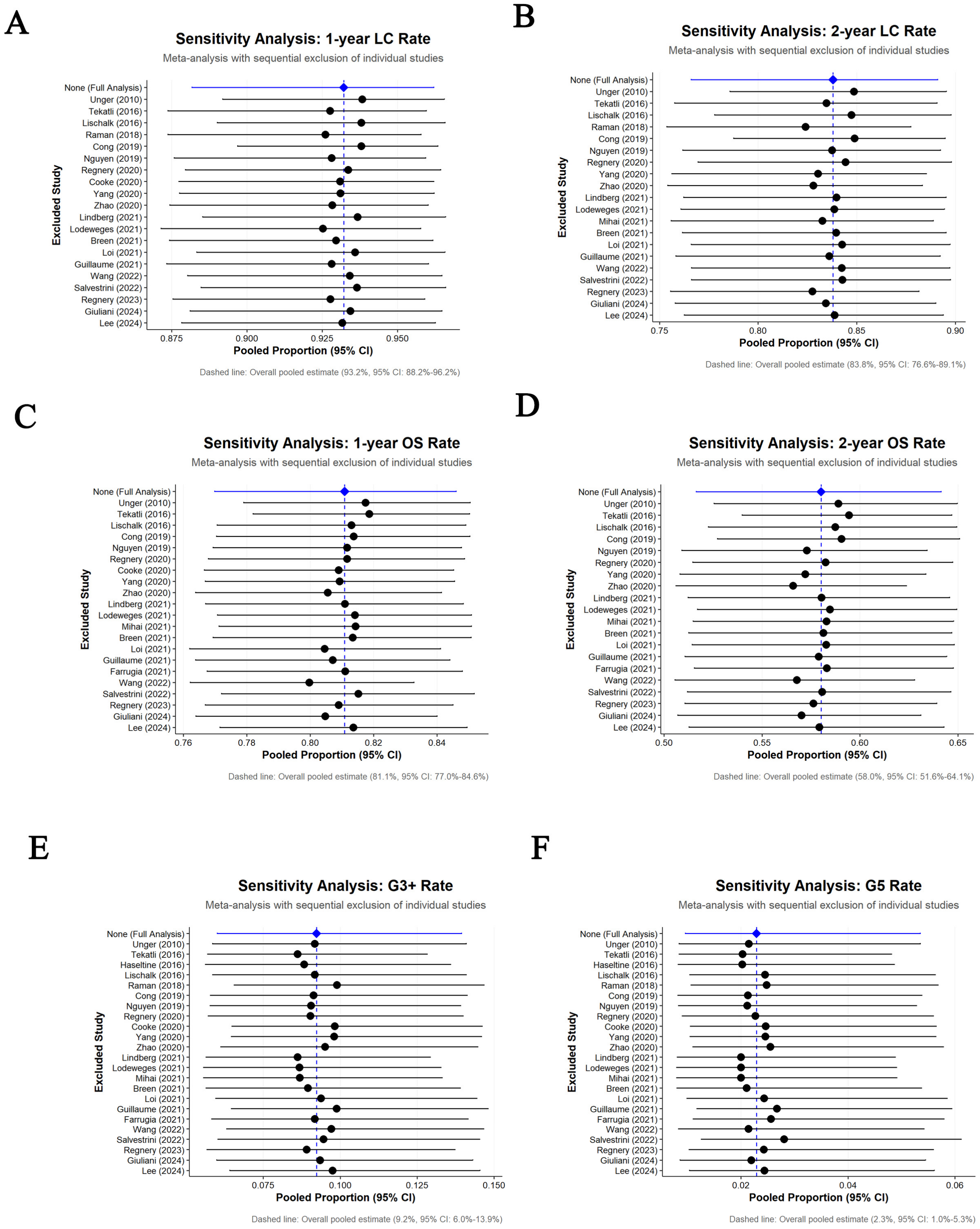

Supplementary Figure 4. Sensitivity analyses of pooled outcomes using the leave-one-out method. Leave-one-out sensitivity analyses were performed to assess the robustness of the pooled estimates by sequentially excluding each individual study. **(A).** 1-year LC rate. **(B).** 2-year LC rate. **(C).** 1-year OS rate. **(D).** 2-year OS rate. **(E).** Grade ≥3 toxicity rate. **(F).** Grade 5 toxicity rate.

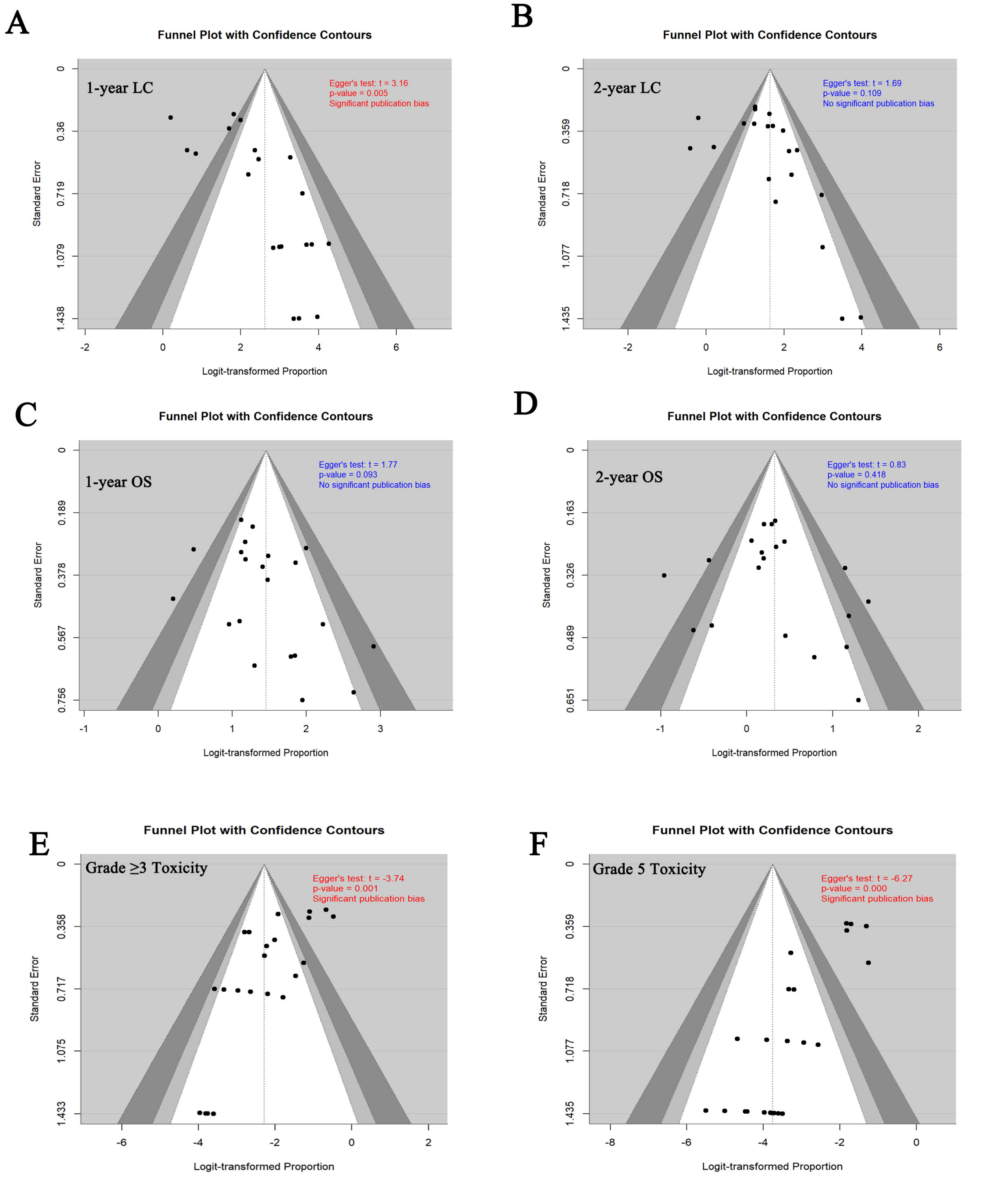

Supplementary Figure 5. Publication bias assessment for key outcomes using funnel plots and Egger's test. **(A).** Funnel plot plots and Egger's test for the pooled 1-year LC rate. **(B).** Funnel plot plots and Egger's test for the pooled 2-year LC rate. **(C).** Funnel plot plots and Egger's test for the pooled 1-year OS rate. **(D).** Funnel plot plots and Egger's test for the pooled 2-year OS rate. **(E).** Funnel plots and Egger's test for the pooled Grade ≥3 toxicity rate. **(F).** Funnel plots and Egger's test for the pooled Grade 5 toxicity rate.

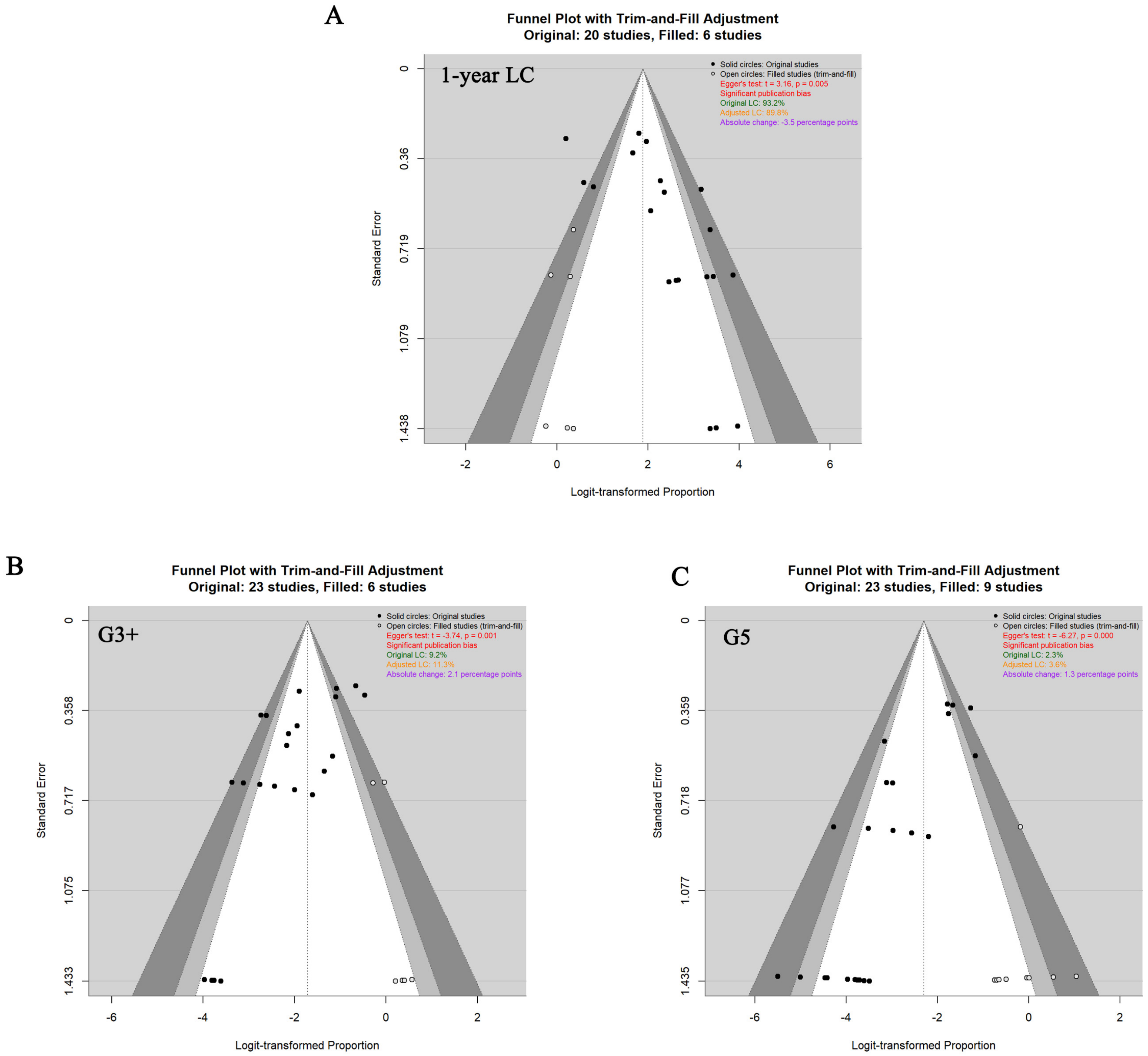

Supplementary Figure 6. Funnel plots with trim-and-fill adjustment for publication bias. **(A)** Funnel plot for the 1-year local control (LC) rate. **(B)** Funnel plot for the grade ≥3 (G3+) toxicity rate. **(C)** Funnel plot for the grade 5 (G5) toxicity rate.

Section and Topic	Item #	Checklist item	Location where item is reported
**TITLE**			
Title	1	Identify the report as a systematic review.	Title Page
**ABSTRACT**			
Abstract	2	See the PRISMA 2020 for Abstracts checklist.	Abstract
**INTRODUCTION**			
Rationale	3	Describe the rationale for the review in the context of existing knowledge.	Introduction, Paragraphs 1-3
Objectives	4	Provide an explicit statement of the objective(s) or question(s) the review addresses.	Introduction, Paragraph 4
**METHODS**			
Eligibility criteria	5	Specify the inclusion and exclusion criteria for the review and how studies were grouped for the syntheses.	Materials and Methods, "Study design" & "Study Selection"
Information sources	6	Specify all databases, registers, websites, organisations, reference lists and other sources searched or consulted to identify studies. Specify the date when each source was last searched or consulted.	Materials and Methods, "Study design" & Supplementary Table 2
Search strategy	7	Present the full search strategies for all databases, registers and websites, including any filters and limits used.	Supplementary Table 2
Selection process	8	Specify the methods used to decide whether a study met the inclusion criteria of the review, including how many reviewers screened each record and each report retrieved, whether they worked independently, and if applicable, details of automation tools used in the process.	Materials and Methods, "Study Selection"
Data collection process	9	Specify the methods used to collect data from reports, including how many reviewers collected data from each report, whether they worked independently, any processes for obtaining or confirming data from study investigators, and if applicable, details of automation tools used in the process.	Materials and Methods, "Study Selection"
Data items	10a	List and define all outcomes for which data were sought. Specify whether all results that were compatible with each outcome domain in each study were sought (e.g. for all measures, time points, analyses), and if not, the methods used to decide which results to collect.	Materials and Methods, "Study endpoints" & "Study Selection"
	10b	List and define all other variables for which data were sought (e.g. participant and intervention characteristics, funding sources). Describe any assumptions made about any missing or unclear information.	Materials and Methods, "Study Selection" & Table 1/2/3
Study risk of bias assessment	11	Specify the methods used to assess risk of bias in the included studies, including details of the tool(s) used, how many reviewers assessed each study and whether they worked independently, and if applicable, details of automation tools used in the process.	Materials and Methods, "Methodological quality assessment"
Effect measures	12	Specify for each outcome the effect measure(s) (e.g. risk ratio, mean difference) used in the synthesis or presentation of results.	Materials and Methods, "Statistical analyses"
Synthesis methods	13a	Describe the processes used to decide which studies were eligible for each synthesis (e.g. tabulating the study intervention characteristics and comparing against the planned groups for each synthesis (item #5)).	Materials and Methods, "Statistical analyses" & Results, "Study descriptions"
	13b	Describe any methods required to prepare the data for presentation or synthesis, such as handling of missing summary statistics, or data conversions.	Materials and Methods, "Study Selection" & "Statistical analyses"
	13c	Describe any methods used to tabulate or visually display results of individual studies and syntheses.	Materials and Methods, "Statistical analyses"
	13d	Describe any methods used to synthesize results and provide a rationale for the choice(s). If meta-analysis was performed, describe the model(s), method(s) to identify the presence and extent of statistical heterogeneity, and software package(s) used.	Materials and Methods, "Statistical analyses"
	13e	Describe any methods used to explore possible causes of heterogeneity among study results (e.g. subgroup analysis, meta-regression).	Materials and Methods, "Statistical analyses"
	13f	Describe any sensitivity analyses conducted to assess robustness of the synthesized results.	Materials and Methods, "Statistical analyses"

Section and Topic	Item #	Checklist item	Location where item is reported
Reporting bias assessment	14	Describe any methods used to assess risk of bias due to missing results in a synthesis (arising from reporting biases).	Materials and Methods, "Statistical analyses"
Certainty assessment	15	Describe any methods used to assess certainty (or confidence) in the body of evidence for an outcome.	Not applicable
**RESULTS**			
Study selection	16a	Describe the results of the search and selection process, from the number of records identified in the search to the number of studies included in the review, ideally using a flow diagram.	Results, "Study descriptions" & Figure 1
	16b	Cite studies that might appear to meet the inclusion criteria, but which were excluded, and explain why they were excluded.	Figure 1
Study characteristics	17	Cite each included study and present its characteristics.	Table 1, Table 2
Risk of bias in studies	18	Present assessments of risk of bias for each included study.	Supplementary Table 3
Results of individual studies	19	For all outcomes, present, for each study: (a) summary statistics for each group (where appropriate) and (b) an effect estimate and its precision (e.g. confidence/credible interval), ideally using structured tables or plots.	Table 2 (LC, OS), Table 3 & Supplementary Table 4 (Toxicity), Forest Plots (Figures 2,3,4)
Results of syntheses	20a	For each synthesis, briefly summarise the characteristics and risk of bias among contributing studies.	Results, "Study descriptions", "Quality assessment", "SBRT delivery"
	20b	Present results of all statistical syntheses conducted. If meta-analysis was done, present for each the summary estimate and its precision (e.g. confidence/credible interval) and measures of statistical heterogeneity. If comparing groups, describe the direction of the effect.	Results, "Local control", "Toxicity", "Overall survival" (Pooled estimates and I^2 reported)
	20c	Present results of all investigations of possible causes of heterogeneity among study results.	Results, "Local control", "Overall survival" (Meta-regression results)
	20d	Present results of all sensitivity analyses conducted to assess the robustness of the synthesized results.	Results, "Local control", "Toxicity", "Overall survival" & Supplementary Figure 4
Reporting biases	21	Present assessments of risk of bias due to missing results (arising from reporting biases) for each synthesis assessed.	Results, "Local control", "Toxicity" & Supplementary Figure 5
Certainty of evidence	22	Present assessments of certainty (or confidence) in the body of evidence for each outcome assessed.	Not applicable
**DISCUSSION**			
Discussion	23a	Provide a general interpretation of the results in the context of other evidence.	Discussion, Paragraphs 1-4
	23b	Discuss any limitations of the evidence included in the review.	Discussion, Paragraph 5
	23c	Discuss any limitations of the review processes used.	Discussion, Paragraph 5
	23d	Discuss implications of the results for practice, policy, and future research.	Discussion, Paragraph 4 & Conclusion
**OTHER INFORMATION**			
Registration and protocol	24a	Provide registration information for the review, including register name and registration number, or state that the review was not registered.	not registered.
	24b	Indicate where the review protocol can be accessed, or state that a protocol was not prepared.	Not reported
	24c	Describe and explain any amendments to information provided at registration or in the protocol.	Not reported

Section and Topic	Item #	Checklist item	Location where item is reported
Support	25	Describe sources of financial or non-financial support for the review, and the role of the funders or sponsors in the review.	Funding section
Competing interests	26	Declare any competing interests of review authors.	Disclosures section
Availability of data, code and other materials	27	Report which of the following are publicly available and where they can be found: template data collection forms; data extracted from included studies; data used for all analyses; analytic code; any other materials used in the review.	Data sharing statement

From: Page MJ, McKenzie JE, Bossuyt PM, Boutron I, Hoffmann TC, Mulrow CD, et al. The PRISMA 2020 statement: an updated guideline for reporting systematic reviews. BMJ 2021;372:n71. doi: 10.1136/bmj.n71
